# Supplementary material for: De novo MYC addiction as an adaptive response of cancer cells to CDK4/6 inhibition
Source: Mol Syst Biol. 2017 Oct 4;13(10):940. doi: 10.15252/msb.20167321 (PMC5658703; doi:10.15252/msb.20167321)
Supplement: Supplementary file 1 — Appendix [file MSB-13-940-s001.pdf]

Table of Contents

1. Appendix Figures
2. Appendix Supplementary Methods
3. Appendix References

1. Appendix Figures

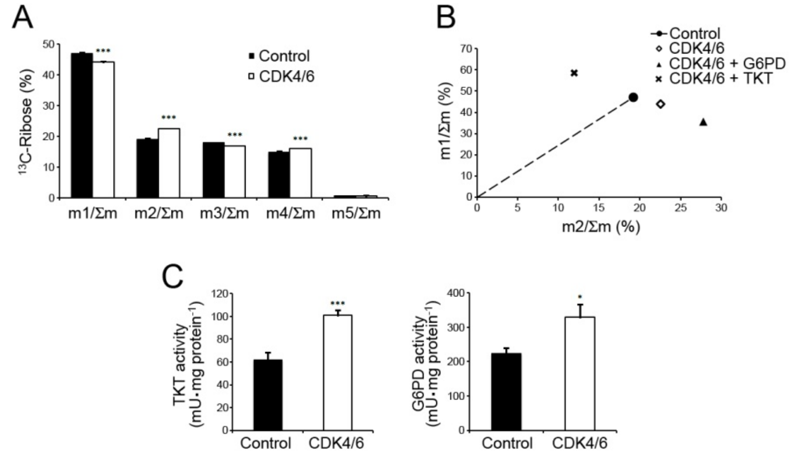

Appendix Figure S1. Related to Figure 1. Pentose phosphate utilization in CDK4/6-kd and control cells. Cells were incubated in the presence of 10 mM [1,2-$^{13}C_2$]-glucose for 24 h. Cell pellets were obtained at this time and ribose was isolated from RNA for isotopologue distribution analysis. (A) Ribose isotopologue distribution normalized to total ^{13}C-Ribose label enrichment (Σm). (B) Isotopologue phase plane analysis for ribose production depicts the contribution of the oxidative and the non-oxidative PPP branches to ribose synthesis. Ribose m1 and m2 were analyzed and plotted as percentages of total ^{13}C-Ribose (Σm). The m2 ribose isotopologues are indicative of the non-oxidative PPP flux, whereas the m1 isotopologues indicate the oxidative PPP flux producing ribose. For clarity, internal controls with combined CDK4/6 and G6PD or TKT siRNA-mediated knockdown were included. (C) Total TKT and G6PD enzyme activities normalized to intracellular protein content. All experiments were performed 96 h after siRNA transfection. CDK4/6, CDK4/6-kd cells; Control, non-targeting siRNA-transfected cells. Data are represented as mean ± SD (n = 3). Statistically significant differences between CDK4/6-kd and control cells are indicated as $p < 0.05$ (*) and $p < 0.001$ (***).

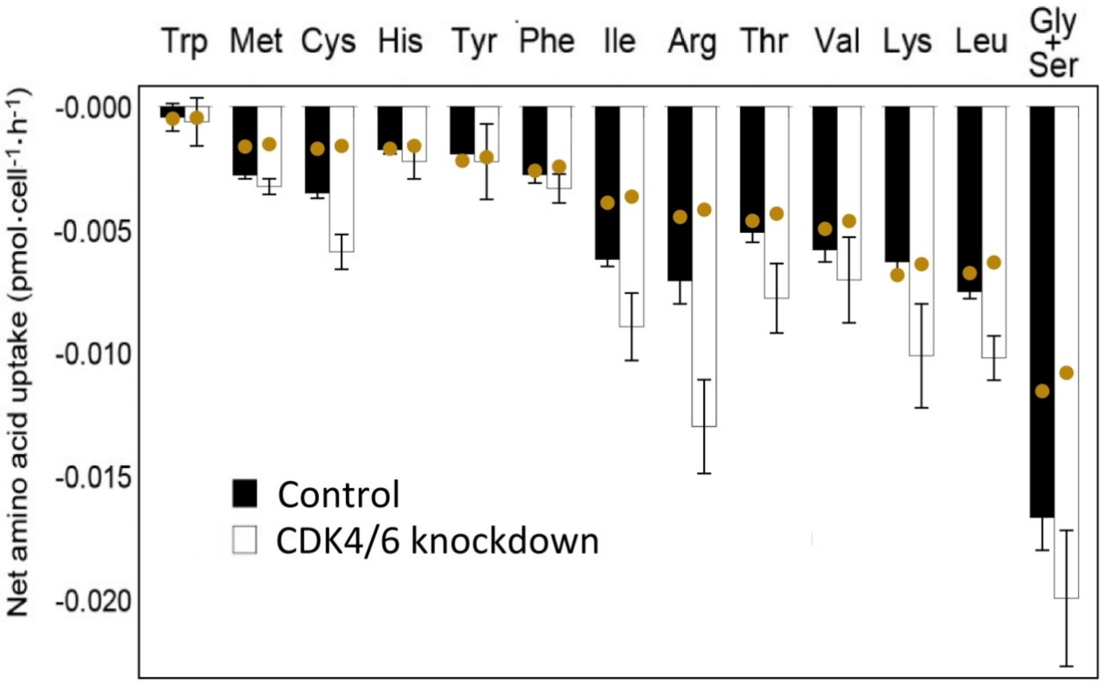

Appendix Figure S2. **Related to Figure 3. Amino acid uptake in CDK4/6-kd and control cells compared with the extra demand of amino acids for protein synthesis.** Bars represent the experimental measurements for amino acid uptake. Golden dots represent the extra demand of a specific amino acid for protein synthesis calculated from the estimated net rate of protein synthesis per amino acid and the relative abundance of that amino acid in proteins (Sheikh et al., 2005).

2. Supplementary Methods

All products were purchased from Sigma-Aldrich Co (St Louis, MO, USA), unless otherwise specified.

Chemicals
PD0332991, 10058-F4, Rapamycin and DMOG were purchased from Sigma-Aldrich. The PI3K inhibitor LY294002 was purchased from Calbiochem (San Diego, CA, USA). The glutaminase inhibitor bis-2-(5-phenylacetamido-1,2,4-thiadiazol-2-yl)ethyl sulfide (BPTES) was kindly provided by Dr Mariia Yuneva (The Francis Crick Institute, London, UK) and CB-839 was purchased from Selleckchem (Houston, TX, USA). In each case, 10 mM stock solutions were prepared with Dulbecco's phosphate buffered saline (PBS), water or dimethyl sulfoxide (DMSO), according to the manufacturers' instructions. Antibiotic (10,000 U/mL penicillin, 10 mg/mL streptomycin), PBS and Trypsin EDTA solution C (0.05% trypsin – 0.02% EDTA) were obtained from Biological Industries (Kibbutz Beit Haemet, Israel), and fetal bovine serum (FBS) from Invitrogen (Carlsbad, CA, USA).

Cell culture
HCT116, SK-BR-3, MCF-7 and BJ cell lines were obtained through the American Type Culture Collection (ATCC, Manassas, VA, USA). NCM460 human epithelial cells derived from the healthy colon mucosa of a 68-year-old Hispanic male (Moyer et al., 1996) were a kind gift from Dr Mary Pat Moyer (INCELL, San Antonio, TX, USA). Phoenix packaging cells were kindly provided by Dr Mariia Yuneva (The Francis Crick Institute, London, UK). All cell lines were regularly tested for mycoplasma contamination. HCT116 human colorectal carcinoma and SK-BR-3 human breast adenocarcinoma cells were cultured in Dulbecco's modified Eagle medium (Gibco, Thermo Fisher Scientific Inc., Waltham, MA, USA) / Nutrient mixture HAM F12 (Biological Industries) (DMEM/F12, 1:1 mixture) with L-glutamine and 12.5 mM D-glucose. BJ human skin fibroblasts and Phoenix cells were grown in DMEM with L-glutamine and 25 mM D-glucose. MCF-7 human breast adenocarcinoma cells were cultured in MEM medium without phenol red (Gibco) containing 10 mM D-glucose, 2 mM L-glutamine, 1 mM pyruvate (Biological Industries), 0.01 mg/mL insulin and 1% non-essential aminoacids (Biological Industries). NCM460 cells were grown in M3Base medium (INCELL) with 5 mM D-glucose and 2 mM L-glutamine. Media were supplemented with 10% heat-inactivated FBS (Invitrogen, Carlsbad, CA), penicillin (50 U/mL) and streptomycin (50 µg/mL). Cells were incubated at 37 ºC in a humidified atmosphere with 5% CO_2. For hypoxia incubations, cells were kept in an atmosphere containing 1% oxygen and 5% CO_2 at 37 ºC in a hypoxia incubator for the indicated time periods.

siRNA transfection
HCT116 cell line was transfected using Lipofectamine RNAiMAX (Invitrogen) transfection reagent. Briefly, 5×10^4 cells/well were seeded in antibiotic-free medium in 6-well flat-bottom tissue culture plates and transfected the next day with a mix (siRNA CDK4/6) containing 30 nM of ON-TARGETplus SMARTpool siRNA against CDK4 (L-003238-00, GE Healthcare Dharmacon Inc., Lafayette, CO, USA) and 30 nM of Silencer Select siRNA against CDK6 (s51, Ambion, Austin, TX, USA) or with a mix (siRNA Control) containing 30 nM of ON-TARGETplus Non-Targeting Control Pool siRNA (D-001810-10, GE Healthcare Dharmacon Inc.) and 30 nM of Silencer Select Negative Control siRNA (4390844, Ambion). For MYC knockdown experiments, cells were transfected with 5 nM Stealth siRNA against MYC (VHS40789, Thermo Fisher Scientific) or 5 nM Stealth siRNA Negative Control (Thermo Fisher Scientific). After 24 h, culture medium was replaced with fresh medium with antibiotics. The CDK4 siRNA pool includes the sequences: CAAGGUAACCCUGGUGUUU, GAGCUCUGCAGCACUCUUA CAGCACAGUUCGUGAGGUG and GCACUUACACCCGUGGUUG. The sequence of Silencer Select siRNA against CDK6 used was GUUUGUAACAGAUAUCGAU. The sequence of MYC Slealth siRNA used was GAGACAUGGUGAACCAGAGUUUCAU. Cells were analyzed 96 h after transfection for CDK4/6 knockdown experiments or 24 h and 96 h after transfection for MYC knockdown assays.

Retroviral transfection and transduction
Phoenix cells were transiently transfected with pBABEpuro-CMYC T58A plasmid (Brady et al., 2014) (a gift from Christopher Counter; Addgene plasmid #53178) for the constitutively expression of the mouse CMYC T58A gene or with pBABE-puro empty vector (Morgenstern and Land, 1990) (a gift from Hartmut Land & Jay Morgenstern & Bob Weinberg; Addgene plasmid #1764) using Fugene reagent (Roche, Indianapolis, IN, USA). Viral supernatants were harvested 48 h and 72 h after transfection, and immediately used to transduce HCT116 recipient cells. 5×10^5 HCT116 cells were cultured in the presence of the viral supernatant and 8 µg/mL polybrene for 24 h and re-infected with fresh viral supernatant and 8

μg/mL polybrene for another 24 h. Transduced cells were selected in complete media containing 2 μg/mL puromycin for 5 days.

Cell proliferation and viability assays

Proliferation assays were performed by flow cytometry combining direct cell counting and propidium iodide (PI) staining. Cells were trypsinised and resuspended in 500 μL of a solution containing 450 μL of complete media, 45 μL of Flow-Count Fluorospheres (Beckman Coulter, Brea, CA, USA) and 5 μL of 1 mg/mL PI. Flow cytometer was adjusted to 1×10^4 fluorospheres cut-off and total cell number was recorded, allowing discrimination between dead and live cells. Cell size and volume values were determined using a Scepter^{TM} Handheld Automated Cell Counter (Merck Millipore, Billerica, MA, USA).

When testing drugs that affect mitochondrial respiration, cell viability was assessed using a Hoechst stain assay (HO33342; 2'-[4-ethoxyphenyl]-5-[4-methyl-1-piperazinyl]-2,5'-bi-1H-benzimidazole trihydrochloride trihydrate). Briefly, 24 h after seeding 2×10^3 cells/well in 96-well plates, media were replaced with complete fresh media containing the desired concentration of drug, the combination of drugs under study, or vehicle. At the end of the experiment, cells were washed with PBS before adding 100 μL of 0.01% SDS per well. Plates were then stored frozen at -20 ºC. To analyze the samples, plates were thawed at 37 ºC until fully liquid and 100 μL of 4 μg/mL HO33342 in stain solution buffer (1 M NaCl, 1 mM EDTA, 10 mM Tris-HCl pH 7.4) added to each well. Tinfoil-covered plates were placed on a shaker and incubated at 37 ºC for 1 h. Fluorescence was quantified on a FLUOstar OPTIMA Microplate Reader (BMG LABTECH GmbH, Ortenberg, Germany) at 355 nm excitation and 460 nm emission. Cell viability was assessed and represented as a percentage of viability relative to untreated control cells.

When testing drugs that interact with DNA, cell viability was assessed with 3-(4,5-dimethylthiazol-2-yl)-2,5-diphenyltetrazolium bromide (MTT) (Mosmann, 1983). Briefly, 2×10^3 cells/well were plated in 96-well-flat-bottomed microtiter plates, allowed to attach for 24 h and test drugs added. After indicated incubation times, 1 mg/mL MTT in PBS was added at a final concentration of 0.5 mg/mL. After 1 hour, supernatants were removed, the formazan product dissolved in 100 μL dimethyl sulfoxide (DMSO) and absorbance measured at 550 nm on a Tecan Sunrise MR20-301 plate spectrophotometer (TECAN, Salzburg, Austria). IC_{50} values were calculated using Graphpad Prism 6 software (La Jolla, CA, USA).

Mitochondrial activity

Total mitochondrial activity per number of cells was estimated by the conversion of the tetrazolium salt MTT into formazan crystals, and direct cell counting of parallel cultures.

Spheroid formation

10^4 cells were grown for 10 days in 24-well ultra-low attachment culture plates (Corning, Corning, NY, USA) in presence of the specified inhibitor(s) in serum-free media supplemented with 20 ng/mL EGF, 20 ng/mL bFGF, 10 μg/mL heparin, B27 (1:50), 5 μg/mL insulin, 0.5 μg/mL hydrocortisone. At the end of the experiment, spheroids were incubated with 0.5 mg/mL MTT for 2-3 h until fully stained. Finally, plates were scanned and spheroids were scored by image acquisition and spheroid area and volume quantification with ImageJ software (public domain National Institutes of Health, USA, http://rsbweb.nih.gov/ij/).

Cell cycle synchronization

Cells were arrested at the cell cycle G1 phase by serum deprivation. Asynchronously growing cells were allowed to reach to approximately 40% confluence, washed with warm PBS and incubated for 48 h with medium without FBS. Cells were collected and processed by flow cytometry and additional assays.

Cell cycle analysis

Cell cycle analysis was performed 96 h after transfection or inhibitor treatment. Both adherent and detached cells were collected by centrifugation after trypsinisation, resuspended in 0.5 mL PBS and added dropwise to 4.5 mL 70% (v/v) cold ethanol. Then, cells were centrifuged, washed with PBS and resuspended in PBS containing 0.2 mg/mL DNAse free RNAse A (Roche, Basel, Switzerland) and incubated for 1 h at 37 ºC. Prior to analysis, 0.05 μg/mL propidium iodide (PI) was added. Flow cytometry analysis was carried out at 488 nm in an Epics XL flow cytometer (Coulter Corporation, Hialeah, FL, USA). Data for 1×10^4 cells were collected and analyzed using the Multicycle program (Phoenix Flow Systems, San Diego, CA, USA), following the cell cycle analysis algorithm described by (Rabinovitch, 1994).

Apoptosis assay

After centrifugation, cells were washed and resuspended in binding buffer (10 mM Hepes pH 7.4, 140 mM sodium chloride, 2.5 mM calcium chloride). Annexin V coupled with fluorescein isothiocyanate (FITC) was added according to the Annexin V-FITC kit's instructions (Bender System MedSystem, Vienna, Austria). Following 30 min incubation at room temperature in the dark, PI was added at 20 µg/mL, 1 min before flow cytometry. Experiments were performed in triplicate and repeated three independent times. Data from 2×10^4 cells were collected and analyzed in each experiment.

Measurement of extracellular metabolites

Glucose, lactate, glutamate and glutamine concentrations from cell culture media were determined using a COBAS Mira Plus spectrophotometer (Horiba ABX, Kyoto, Japan) to monitor the production of NAD(P)H in specific reactions for each metabolite at 340 nm. Glucose concentration was measured using hexokinase (HK) and glucose-6-phosphate dehydrogenase (G6PD) coupled enzymatic reactions (ABX Pentra Glucose HK CP, HORIBA ABX, Montpellier, France). Lactate concentration was determined by lactate dehydrogenase (LDH) reaction at 37 ºC by mixing the media samples with 1.55 mg/mL NAD^+ and 87.7 U/mL LDH (Roche) in 0.2 M hydrazine 12 mM EDTA buffer (pH 9). Glutamate concentration was assessed by conversion to α-ketoglutarate through glutamate dehydrogenase (GDH) reaction in the presence of ADP at 37 ºC by adding media samples to 2.41 mM ADP, 3.9 mM NAD^+ and 39 U/mL of GDH (Roche) in 0.5 M glycine/0.5 M hydrazine buffer (pH 9). Glutamine concentration was calculated by means of its conversion to glutamate by glutaminase (GLS) and subsequent quantification of glutamate concentration as above. The GLS reaction was carried out by incubating the media samples with 125 mU/mL GLS in 125 mM acetate buffer (pH 5) for 30 min at 37 ºC.

Concentrations of non-essential and essential amino acids (alanine, aspartate, asparagine, proline, glycine, serine, arginine, cysteine, threonine, isoleucine, leucine, lysine, methionine, valine, tryptophan, histidine, phenylalanine, tyrosine, glutamate and glutamine) in cell media were determined by ion-exchange chromatography with a Biochrom 30 amino acid analyzer (Pharmacia Biochrom Ltd, Cambridge, UK). As an internal standard, 70 µL of 150 µM norleucine were added to 500 µL of medium. Then, samples were dried by SpeedVac (Thermo Fisher Scientific Inc.), resuspended in 500 µL lithium citrate buffer (pH 2.2) and filtered through a 0.22 µm filter. 30 µL of each sample were injected into the Biochrom 30 lithium system. A set of lithium citrate buffers were used as mobile phase for separation during 115 min and post column derivatization with ninhydrin allowed amino acid detection at 570 and 440 nm. The retention time of the peak on the chart allowed identification of amino acids and the area under the peak correlated with the quantity of amino acid.

In order to calculate the consumption/production rate of each metabolite, media samples were collected at the beginning and end of the experiment and frozen until analyzed. At the same time points, cell numbers were determined for normalization. All biochemical assays were carried out under exponential growth conditions. All results are expressed in micromol or nanomol of metabolite consumed or produced per hour and per million cells.

Estimation of metabolite consumption and production rates

Net fluxes per cell of uptake and release of different metabolites (J_{met}) were estimated from the experimentally measured variations of metabolite concentration in medium and cell number for 24 h. The estimation was performed by assuming exponential growth and constant uptake or release per cell, which corresponds to a simple model of cell growth and metabolite consumption/production:

$$\begin{cases} dN_t/dt = N_t \times \mu \\ dM_t/dt = N_t \times J_{met} \end{cases}$$

where N is cell number, M quantity of metabolite and μ growth rate. This simple model for flux estimation was verified to be a good assumption for the experimental period of time and is equivalent to the model used to measure the consumption and release profiles of 219 medium metabolites across the NCI-60 panel of tumor-derived cell lines (Jain et al., 2012), which include the HCT116 cell line. Our exchange rates were in tune with those measured for the NCI-60 collection.

Enzymatic activities
Cells were rinsed with PBS, incubated for 30 min at 4 ºC with lysis buffer (20 mM Tris-HCl pH 7.5, 1 mM dithiothreitol, 1 mM EDTA, 0.2% Triton X-100, 0.02% sodium deoxycholate) supplemented with 1% protease inhibitor cocktail, scraped and collected in 1.5 mL eppendorf tubes. Cell lysates were disrupted by sonication using a titanium probe (VibraCell, Sonics & Materials Inc., Newtown, CT, USA) set at Tune 50 and Output 30, and immediately centrifuged at 12,000 g for 20 min at 4 ºC. Supernatants were recovered and the protein content was quantified by the bicinchoninic acid (BCA) procedure (Pierce Biotechnology, Rockford, IL, USA). Specific enzyme activities were determined by spectrophotometry (COBAS Mira Plus, Horiba ABX) by monitoring NAD(P)H increment or decrement at 340 nm. Enzymatic activities were normalized by protein content in the supernatant.

Glucose-6-phosphate dehydrogenase (G6PD, EC 1.1.1.49)
G6PD specific activity was determined by incubation of the protein extracts with 0.5 mM $NADP^+$ in 50 mM Tris-HCl (pH 7.6) at 37 ºC. The reaction was initiated by addition of glucose-6-phosphate at a final concentration of 2 mM.

Transketolase (TKT, EC 2.2.1.1)
TKT specific activity was measured by incubation of the protein extracts with 5 mM $MgCl_2$, 0.2 U/mL triose phosphate isomerase, 0.2 mM NADH and 0.1 mM thiamine pyrophosphate in 50 mM Tris-HCl (pH 7.6) at 37 ºC. The reaction was initiated by addition of a substrate mixture containing ribose-5-phosphate and xylulose-5-phosphate. The substrate mixture was previously prepared by dissolving 50 mM ribose-5-phosphate in 50 mM Tris-HCl (pH 7.6) in the presence of 0.1 U/mL ribulose-5-phosphate-3-epimerase and 1.7 mU/mL phosphoriboisomerase in continuous agitation at 37 ºC for 1 h.

Pyruvate dehydrogenase (PDH, EC 1.2.4.1) activity
PDH activity was measured with the Pyruvate dehydrogenase (PDH) Enzyme Activity Microplate Assay kit (Abcam, Cambridge, UK) according to the manufacturer's instructions. Briefly, cells were collected, counted and resuspended in PBS to adjust the sample protein concentration to 15 mg/mL. Then, samples were solubilized with Detergent (9:1), incubated on ice for 10 min and centrifuged at 1,000 g for 10 min at 4 ºC. The supernatants were diluted in Assay Buffer to the appropriated concentration within the linear working range for the assay, loaded to each well of the microplate and incubated at room temperature for 3 h. For the measurement, wells were emptied and 200 μL of Assay Solution were added. The absorbance was measured at 450 nm using a kinetic program with readings every 25 sec for 30 min. PDH activity was normalized by protein content in the supernatant, determined by the bicinchoninic acid (BCA) assay.

Intracellular glutathione quantification
Total glutathione content was determined by the glutathione reductase enzymatic method. Fresh cells were lysed with 5% 5-sulfosalicylic acid solution, vortexed and disrupted by two freezing/thawing cycles in liquid N_2 and 37 ºC water bath. For each sample, 50 μL of cell lysate were separated for subsequent protein quantification by BCA assay. Cell extracts were incubated at 4 ºC for 10 min and centrifuged at 10,000 g for 10 min. For glutathione quantification, a working solution containing 15 U/mL of glutathione reductase and 40 μg/mL of 5,5'-Dithiobis(2-nitrobenzoic acid) was prepared in assay buffer (100 mM K2HPO4/KH2PO4, 1 mM EDTA, pH 7.0). Glutathione standards were prepared from a 50 mM oxidized glutathione (GSSG) stock solution. The reaction was initiated by mixing 150 μL working solution with 10 μL cell extract (diluted 1:5 or 1:10) or 10 μL GSSG standard (final concentrations from 0 to 12.5 μM). Next, 50 μL 0.16 mg/mL NADPH solution were added to the samples and the absorbance was recorded at 340 nm. Total glutathione concentration was normalized by protein content and cell number.

Intracellular NADP and NADPH quantification
Intracellular NADP and NADPH were quantified using the NADP/NADPH Quantification Kit (MAK038, Sigma-Aldrich), following the manufacturer's instructions. Briefly, fresh cells were washed twice with ice-cold PBS, trypsinized and lysed with 200 μL NADP/NADPH Extraction Buffer by two freeze/thawing cycles of 20 min on dry ice followed by 10 min at room temperature. Then, samples were vortexed and centrifuged at 13,000 g for 10 min. For the detection of total NADP, 50 μL of supernatant were transferred to a 96-well plate. To detect only NADPH, NADP was first decomposed by incubating 200 μL of the extracted samples at 60 ºC for 30 min before transferring 50 μL of sample to the 96-well plate. For NADPH quantification, a standard curve was generated of 0 (blank), 20, 40, 60, 80, and 100 pmol NADPH per well. 100 μL of Master reaction Mix containing 98 μL NADP cycling buffer and 2 μL NADP cycling enzyme Mix were added to each well, mixed and incubated for 5 min at room temperature (to convert NADP to NADPH). Then, 10 μL NADPH developer were added to each well and samples

incubated for 1 to 4 h at room temperature and absorbance measured at 450 nm. NADP and NADPH calculated concentrations were normalized by cell number.

Intracellular reactive oxygen species (ROS) levels

Cells were grown on 6-well plates to 70% confluence, washed once with warm PBS, and incubated with 5 μM 2'-7'-dichlorodihydrofluorescein diacetate (H_2DCFDA, Invitrogen) in PBS supplemented with 5.5 mM glucose. After 30 min at 37 ºC, PBS was replaced with complete culture medium and incubated for another 50 min at 37 ºC. Finally, cells were trypsinized and resuspended thoroughly with 0.4 mL of PBS, H_2DCFDA (50 μM) and PI (20 μg/mL). The emitted fluorescence was recorded by flow cytometry at 520 nm using an Epics XL flow cytometer (Coulter Corporation, Hialeah, FL, USA). Data for DCF fluorescence intensity from 1×10^4 PI negative cells were collected and analyzed using Multicycle program (Phoenix Flow Systems, San Diego, CA, USA).

Western blotting

Cells were washed twice with ice-cold PBS and incubated for 30 min on ice with RIPA buffer containing 50 mM Tris (pH 8.0), 150 mM sodium chloride, 1% Triton X-100, 0.5% sodium deoxycholate, 0.1% sodium dodecyl sulphate (SDS), 1% protease inhibitor cocktail and 1% phosphatase inhibitor cocktail (Thermo Fisher Scientific Inc.). Cells were scraped, sonicated and centrifuged at 16,000 g for 20 min at 4 ºC. Supernatants were recovered and the protein content was quantified by the BCA kit (Pierce Biotechnology). Equal amounts of protein per sample were size-separated by electrophoresis on SDS-polyacrylamide gels and electroblotted onto polyvinylidene fluoride transfer membranes (PVDF) (Bio-Rad Laboratories, Hercules, CA, USA). After 1 h of blocking at room temperature with 5% skim milk in PBS 0.1% Tween, blots were incubated with the specific primary antibodies overnight at 4 ºC. Then, membranes were treated with the appropriate secondary antibody for 1 h at room temperature. All blots were treated with Immobilon ECL Western Blotting Detection Kit Reagent (EMD Millipore, Billerica, MA, USA) and developed after exposure to an autoradiography film (VWR International, Radnor, PA, USA). The primary antibodies used were Phospho-Akt (Ser473) (#9271), Akt (#9272), Phospho-p70 S6 Kinase (Thr389) (#9205), p70 S6 Kinase (#9202), Phospho-mTOR (Ser2448) (#5536), mTOR (#2972), HK2 (#2867) and PDK1 (#3820) from Cell Signaling (Beverly, MA, USA); PDH (ab110330), GLS1 (ab93434), c-MYC (ab32072), c-MYC (phospho S62) (ab106952), c-MYC (phospho T58) (ab28842), PHD2 (ab4561) and ME2 (ab139686) from Abcam; P-PDH (ABS204) from Millipore (EMD Millipore); GAC (19958-1-AP) and KGA (20170-1-AP) from Proteintech (Chicago, IL, USA); CDK4 (sc-260), CDK6 (sc-177), HIF1α (sc-13515), EPAS-1 (HIF2α, sc-28706), TSC1 (hamartin) (sc-12082), TSC2 (tuberin) (sc-893) and Ub (sc-8017) from Santa Cruz Biotechnology; GDH (GTX105765) from Tebu-Bio (Le-Perray-en-Yvelines, France); $p27^{Kip1}$ (#610242) from BD Transduction Laboratories (BD Biosciences) and β-actin (#69100) form MP Biomedicals (Santa Ana, CA, USA). The secondary antibodies used were anti-mouse (PO260) from Dako (Glostrup, Denmark), anti-rabbit (NA934V) from Amersham Biosciences (GE Healthcare, Little Chalfont, UK) and anti-goat (sc-2020) from Santa Cruz Biotechnology.

Immunoprecipitation

Whole cell lysates were isolated as described above, pre-cleared adding Protein A agarose beads (20 μL of 50% bead slurry; Cell Signaling) to 200 μL cell lysate and incubating on a rotator at 4 ºC for 1 h. Then, samples were spinned for 10 min at 4 ºC and the supernatant protein concentration determined with the BCA kit. For each sample, 200 μg of protein were incubated with 1 μg of anti-c-MYC antibody (ab32072, Abcam) with gentle rocking overnight at 4 ºC. Protein immunocomplexes were then incubated with 20 μL of 50% bead slurry protein A agarose beads with gentle rocking for 3 h at 4 ºC, collected by centrifugation (30 sec at 4 ºC) and washed five times in 500 μL of RIPA buffer containing 1% protease and 1% phosphatase inhibitors. The pellet was resuspended with 20 μL 3X SDS loading buffer, vortexed and centrifuged for 30 sec. Finally, samples were heated to 100 ºC for 5 min, centrifuged for 1 min at 14,000 g and loaded on a SDS-PAGE gel for Western blotting.

RNA extraction, quantification, retrotranscription and Quantitative Reverse Transcription-Polymerase Chain Reaction (qRT-PCR)

Cells were rinsed with PBS and plates frozen. RNA was isolated using Trizol reagent (Invitrogen) following. Briefly, Trizol cell homogenates were mixed with chloroform and centrifuged, obtaining an aqueous phase and an organic phase. Cold isopropanol was added to the aqueous phase and centrifuged at 12,000 g for 15 min at 4 ºC. RNA was purified by several cold 75% ethanol washes and finally resuspended in RNAse free water. RNA was quantified using a Nanodrop spectrophotometer (ND 1000 V3.1.0, Thermo Fisher Scientific Inc.). Reverse transcription was carried out with 1 μg RNA at 37 ºC for 1 h with the following reagents: 5x Buffer (Invitrogen), 0.1 M dithiothreitol (DTT) (Invitrogen), Random

Hexamers (Roche), 40 U/µL RNAsin (Promega, Fitchburg, WI, USA), 40 mM dNTPs (Bioline, London, UK), 200 U/µL M-MLV-RT (Invitrogen). Gene expression analysis was performed on an Applied Biosystems 7500 Real-Time PCR System according to the manufacturer's protocol, using Taqman gene specific sequences for *CCND1* (Hs00765553_m1), *CDK4* (Hs00262861_m1), *CDK6* (Hs01026371_m1), *CDKN1B* (Hs01597588_m1), *EGLN1* (Hs00254392_m1), *ENO2* (Hs00157360_m1), *FOXO1* (Hs01054576_m1), *FOXO3a* (Hs00818121_m1), *G6PD* (Hs00166169_m1), *GLS1* (Hs01014019_m1), *GSK3B* (Hs01047719_m1), *HK2* (Hs00606086_m1), *IDH2* (Hs00158033_m1), *MAX* (Hs04332980_m1), *ODC1* (Hs00159739_m1), *PDK1* (Hs01561850_m1), *PDK3* (Hs00178440_m1), *PFKFB4* (Hs00190096_m1), *PIK3R3* (Hs00177524_m1), *SAT1* (Hs00161511_m1), *SLC2A3* (Hs00359840_m1), *SLC2A6* (Hs01115485_m1), *SLC3A2* (Hs00374243_m1), *SLC7A5* (Hs00185826_m1), *SLC7A6* (Hs00938056_m1), *SLC25A13* (Hs01573628_m1) and *TKT* (Hs00169074_m1) (Applied Biosystems, Thermo Fisher Scientific Inc.). Reactions were performed in a final volume of 20 µL, containing 9 µL of cDNA mixture and 11 µL of the specific Taqman in Master Mix (Applied Biosystems). Real-Time PCR was conducted according to the following parameters: an initial incubation at 50 ºC for 2 min and denaturalization at 95 ºC for 10 min, followed by 40 cycles at 95 ºC and 60 ºC for 15 sec and 1 min, respectively. Expression was quantified by ΔΔCt method using Cyclophilin A (*PPIA*: Hs99999904_m1, Applied Biosystems) as reference gene.

Kinase assay

Kinase assays were performed at Reaction Biology Corp. (Malvern, PA, USA) using the "HotSpot" assay platform (Anastassiadis et al., 2011). In brief, recombinant human c-MYC protein (Abcam, ab169901) was prepared along with required cofactors in freshly prepared Base Reaction Buffer (20 mM Hepes pH 7.5, 10 mM $MgCl_2$, 1 mM EGTA, 0.02% Brij35, 0.02 mg/ml BSA, 0.1 mM Na_3VO_4, 2 mM DTT, 1% DMSO). Then, CDK4-Cyclin D1 or CDK6-Cyclin D1 complexes were delivered into the substrate solution at varying concentrations (kinase titration) and gently mixed. A kinase titration without substrate was also performed to determine the background signal, and Retinoblastoma protein was used as a substrate to obtain a positive phosphorylated control. ^{33}P-ATP was added into the reaction mixture to initiate the reaction and incubated for 60 and 120 min at room temperature. Reactions were spotted onto P81 ion exchange filter paper (Whatman Inc., Piscataway, NJ, USA; #3698-915). Unbound phosphate was removed by washing filters extensively in 0.75% phosphoric acid. ^{33}P signal was determined using Typhoon phosphorimagers (GE Healthcare). For mass spectrometry phosphorylation site profiling and western blot, cold ATP was used instead of ^{33}P-ATP.

Mass spectrometry phosphorylation site profiling

Mass spectrometry phosphosite profiling was performed at the CRG/UPF Proteomics Unit. Samples were reduced with dithiothreitol (6 nmol, 30 min, 56 °C), alkylated in the dark with iodoacetamide (12 nmol, 30 min, 25 ºC) and digested with 0.2 µg of trypsin (Promega, #V5113) overnight. After digestion, the peptide mix was acidified with formic acid and desalted with a MicroSpin C18 column (The Nest Group, Inc) prior to LC-MS/MS analysis (Wisniewski et al., 2009).

The peptide mixes were analyzed using a LTQ-Orbitrap Velos Pro mass spectrometer (Thermo Fisher Scientific, San Jose, CA, USA) coupled to an EasyLC (Thermo Fisher Scientific (Proxeon), Odense, Denmark). Peptides were loaded onto the 2-cm Nano Trap column with an inner diameter of 100 µm packed with C18 particles of 5 µm particle size (Thermo Fisher Scientific) and were separated by reversed-phase chromatography using a 25-cm column with an inner diameter of 75 µm, packed with 1.9 µm C18 particles (Nikkyo Technos Co., Ltd. Japan). Chromatographic gradients started at 93% buffer A and 7% buffer B with a flow rate of 250 nl/min for 5 minutes and gradually increased 65% buffer A and 35% buffer B in 60 min. After each analysis, the column was washed for 15 min with 10% buffer A and 90% buffer B. Buffer A: 0.1% formic acid in water. Buffer B: 0.1% formic acid in acetonitrile. As a quality control, BSA controls were run between each sample to avoid carryover and assess the instrument performance.

The mass spectrometer was operated in DDA mode and full MS scans with 1 micro scans at resolution of 60.000 were used over a mass range of m/z 350-2000 with detection in the Orbitrap. Auto gain control (AGC) was set to 1E6, dynamic exclusion (60 seconds) and charge state filtering disqualifying singly charged peptides was activated. In each cycle of DDA analysis, following each survey scan the top twenty most intense ions with multiple charged ions above a threshold ion count of 5000 were selected for fragmentation at normalized collision energy of 35%. Fragment ion spectra produced via collision-induced dissociation (CID) were acquired in the Ion Trap, AGC was set to 5e4, isolation window of 2.0 m/z, activation time of 0.1 ms and maximum injection time of 100 ms was used. All data were acquired with Xcalibur software v2.2.

Data Analysis

Proteome Discoverer software suite (v1.4, Thermo Fisher Scientific) and the Mascot search engine (v2.5, Matrix Science (Perkins et al., 1999)) were used for peptide identification and phosphoRS (v3.0) for phophosite localization. Samples were searched against a Swiss-Prot human database containing c-Myc (P01106-2) plus a list of common contaminants and all the corresponding decoy entries (20720 entries). Trypsin was chosen as enzyme and a maximum of three miscleavages were allowed. Carbamidomethylation (C) was set as a fixed modification, whereas oxidation (M), acetylation (N-terminal) and phosphorylation (STY) were used as variable modifications. Searches were performed using a peptide tolerance of 7 ppm, a product ion tolerance of 0.5 Da. Resulting data files were filtered for FDR < 5 %. Protein top 3 areas were calculated with unique peptides per protein.

Transcriptomic analyses

Microarray analysis (Affymetrix U133 Plus 2.0 array)

The integrity of RNA was determined on a BioAnalyzer 2100 instrument (Agilent, Palo Alto, CA). RNA was used to produce biotinylated cRNA that was hybridized to Affymetrix GeneChip® human genome U133 Plus 2.0 arrays (Affymetrix Inc., Santa Clara, CA, USA). Comparative transcriptomic analyses between CDK4/6 knockdown and control cells were performed on independent triplicate samples. The microarray data have been deposited in NCBI's Genome Expression Omnibus (GEO) database (Edgar et al., 2002) and are accessible through GEO series accession number GSE84597 (https://www.ncbi.nlm.nih.gov/geo/query/acc.cgi?acc=GSE84597). Files with a .CEL extension were uploaded to the R-Project Bioconductor statistical tools package and standardized using the Robust Multi-array Average (RMA) method (Irizarry et al., 2003). We used the simpleaffy (Wilson and Miller, 2005) package to compute RMA expression values (signal intensities on a base 2 logarithm scale and differential gene expression was assessed using the LIMMA (Smyth, 2004) package from Bioconductor. Multiple testing adjustment of p-value was conducted according to Benjamini-Hochberg FDR correction (Benjamini and Hochberg, 1995). Fold change (FC) and Log_2 fold changes (log_2FC) values are provided to illustrate differential expressions for each probe set.

Gene association studies

Gene Set Enrichment Analysis (GSEA) (Subramanian et al., 2005) was applied to infer gene signatures significantly associated with differentially expressed genes. GSEA was applied to analyze our computed RMA expression values over four data sets - Hallmark gene sets, Curated gene sets, GO gene sets, and Oncogenic signatures - created by merging signatures downloaded from the Molecular Signatures Database (MSigDB v5.0) (Subramanian et al., 2005), by selecting the following options: True for "Collapse data set to symbols", Gene set for "Permutation type", 1000 for "Number of permutations", Weighted for "Enrichment statistic", Signal2Noise for "Metric for ranking genes", Real for "Gene list sorting mode", Descending for "Gene list ordering mode", 500 for "Max size: exclude larger sets" and 15 for "Min size: exclude smaller sets". A normalized enrichment score (NES) was used to rank the enriched gene sets in each phenotype (control vs. CDK4/6 knockdown). A false discovery rate q-value (FDR q-value) was also computed in order to estimate the probability that a gene set with a given NES was representing a false positive finding. Finally, a family wise-error rate p value (FWER p value) was computed in order to estimate the probability that the normalized enrichment score represents a false positive finding. Gene sets with a FDR q value ≤ 5% were considered significant. Results are included in Table EV3.

Selection of differentially expressed genes

Differentially expressed genes were identified from normalized RMA expression data obtained from the Affymetrix GeneChip arrays comparing CDK4/6 knockdown and control cells. As a preliminary criteria the selection was restricted to those probe-sets with a $|FC| > 1.5$ ($|log_2 FC| > 0.585$) and a p-adjusted value lower than 0.05. Probe sets satisfying these criteria are provided in Table EV2.

^{13}C-tracer-based metabolomics. ^{13}C labeling patterns

In order to obtain precise labeling patterns of central carbon metabolism, cells were incubated in the presence of either 10 mM [1,2-$^{13}C_2$]-glucose or 2 mM [U-$^{13}C_5$]-glutamine for 24 h. After a 24 h incubation, media and cell cultured plates were collected and stored at -20 ºC and -80 ºC, respectively. Media samples were used for the determination of isotopologue (mass isotopomer) distributions of glucose, lactate, glutamate/glutamine, alanine, aspartate/asparagine, proline and serine. Cell extracts were used for the analysis of isotopologue distributions of glycogen, RNA ribose, fatty acids and internal metabolites including pyruvate, lactate, alanine, aspartate, glutamate and tricarboxylic acid (TCA) cycle intermediates. Isotopologue distribution analyses of ^{13}C-labeled extracellular and intracellular metabolites

were conducted by gas chromatography coupled to mass spectrometry (GC/MS) using an Agilent 7890A gas chromatographer (Agilent Technologies, Santa Clara, CA, USA) equipped with a HP-5 capillary column connected to an Agilent 5975C mass spectrometer (Agilent). Fatty acids GC/MS analysis was performed employing a GCMS-QP 2012 Shimadzu instrument (Shimadzu Corporation, Kyoto, Japan) equipped with a BPX70 column (SGE Analytical Science, Melbourne, Australia). In all cases, 1 μL of sample was injected using helium as a carrier gas at a flow rate of 1 mL/min. Metabolite isolation and derivatization methods as well as GC/MS detection conditions are summarized in Table EV1.

Glucose

Glucose was isolated from cell culture media using a tandem set of Dowex-1X8/Dowex-50WX8 ion-exchange columns, with water elution. Then, water was evaporated to dryness under airflow overnight. Isolated glucose was derivatized in two steps: incubation with 100 μL 2% (v/v) hydroxylamine hydrochloride in pyridine for 30 min at 100 ºC and then with 75 μL acetic anhydride for 60 min at 100 ºC. Excess reagent and solvent were eliminated by evaporation under N_2 flow, and glucose derivative was dissolved in ethyl acetate for GC/MS analysis under chemical ionization mode. Samples were injected at 250 ºC and the oven temperature was programmed as follows: 230 ºC for 2 min, then increased at 10 ºC/min to 260 ºC, followed by a 25 ºC/min ramp to 270 ºC and hold for 2 min. The detector was run in SIM, recording ion abundance of C1-C6 molecule in the range of 327-334 m/z. The retention time was 3.7 min.

Lactate

For each sample, 1 mL of medium was acidified with hydrochloric acid (HCl) and lactic acid was extracted with 1 mL ethyl acetate and evaporated to dryness under N_2 flow. Lactate was derivatized to its lactic acid n-propylamide-heptafluorobutyric ester by incubation at 75 ºC for 1 h with 200 μL of 2,2-dimethoxypropane and 50 μL of 0.5 N methanolic HCl. 60 μL of n-propylamine were added to the reaction mixture and heated at 100 ºC for 1 h. Samples were dried under a stream of N_2. Derivative products were extracted with 1 mL of ethyl acetate and filtered through a glass wool-packed Pasteur pipette. Samples were dried under N_2 flow. Then, precipitates were resuspended with 200 μL of dichloromethane and 15 μL of heptafluorobutyric anhydride at room temperature for 10 min. Samples were dried under N_2 flow and resuspended in dichloromethane for GC/MS analysis under chemical ionization mode. Samples were injected at 200 ºC and the oven temperature was programmed as follows: 100 ºC for 3 min, then increased at 20 ºC/min to 160 ºC and held for 2 min. The detector was run in SIM recording ion abundance of C1-C3 molecule in the range of 327-332 m/z. The retention time was 5.4 min.

Glutamate/Glutamine

Media were passed through Dowex-50WX8 columns and amino acids were eluted with 10 mL of 2 N ammonium hydroxide. Eluates were left to dry under airflow overnight and then resuspended with 5 mL of Milli-Q water. Columns were washed with water and glutamate was collected with 10 mL of 0.5 N acetic acid. The acid solutions were evaporated to dryness under airflow overnight. Glutamate was converted to its n-trifluoroacetyl-n-butyl ester by incubation with 200 μL of butanolic HCl at 100 ºC for 1 h. Then, samples were dried under a stream of N_2 and 100 μL of dichloromethane and 25 μL of trifluoroacetic anhydride were added. After 20 min, samples were dried under a N_2 flow and the derivative was dissolved in dichloromethane for GC/MS analysis under electron impact mode, yielding C2-C5 glutamate fragments. Samples were injected at 250 ºC and the oven temperature was programmed as follows: 215 ºC for 2 min, then increased at 9 ºC/min to 224 ºC and at 3 ºC/min to 233 ºC and held for 2 min. The detector was run in SIM mode recording ion abundance in the range of 197-203 m/z for C2-C5 fragments. The retention time was 3.9 min.

Alanine, aspartate/asparagine, glutamate/glutamine, proline and serine

Media were passed through Dowex-50WX8 columns and amino acids were eluted with 10 mL of 2 N ammonium hydroxide. Solutions were then evaporated to dryness under airflow overnight. Amino acids were converted to their n-trifluoroacetyl-n-butyl ester by incubation with 200 μL of butanolic HCl at 100 ºC for 1 h. Excess reagent was removed under a stream of N_2 and the precipitate was dissolved in 100 μL of dichloromethane and 25 μL of trifluoroacetic anhydride at room temperature for 20 min. Then, samples were dried under a stream of N_2 and dissolved in dichloromethane for GC/MS analysis under chemical ionization mode. Samples were injected at 250 ºC and the oven temperature was programmed as follows: 110 ºC for 1 min, then increased at 10 ºC/min to 125 ºC, 5 ºC/min to 153 ºC, 50 ºC/min to 200 ºC, 5 ºC/min to 216 ºC and held for 1 min, and a final 25 ºC/min ramp to 250 ºC and held for 2 min. The detector was run in SIM mode recording ion abundance in the range of 241-246 m/z for C1-C3 alanine (Retention time, RT: 5.3 min), 341-348 m/z for C1-C4 aspartate and asparagine (RT: 11.5 min), 383-390

m/z for C1-C5 glutamate/glutamine (RT: 12.8 min); 295-302 for C1-C5 proline (RT: 9.6 min) and 353-358 for C1-C3 serine (RT: 6.6 min).

Ribose

Ribose from RNA was isolated from the aqueous phase after addition of Trizol reagent to cell cultured plates as described above. Purified RNA was hydrolyzed in 2 mL of 2 N HCl at 100 ºC for 2 h and the solvent was evaporated to dryness under airflow overnight. RNA ribose was converted to its ribose aldonitrile acetate derivative after treatment at 100 ºC for 30 min with 100 µL of hydroxylamine hydrochloride in pyridine (2% v/v) and then at 100 ºC for 1 h with 75 µL of acetic anhydride. Excess reagent and solvent were removed by evaporation under N_2 flow, and the derivatized ribose was resuspended in ethyl acetate just before GC/MS analysis under chemical ionization mode. Samples were injected at 250 ºC and the oven temperature was programmed as follows: 150 ºC for 1 min, then increased at 15 ºC/min to 275 ºC and finally to 300 ºC at 40 ºC/min. Detection was run in SIM recording ion abundance of C1-C5 molecule in the range of 255-262 m/z. The retention time was 5.3 min.

Fatty acids: palmitate and stearate

Fatty acids from cell cultured plates were hydrolyzed from the inter- and organic phase obtained from the Trizol extract as described above, by adding 500 µL of 100% ethanol and 300 µL of 30% potassium hydroxide. Samples were then incubated at 70 ºC overnight, after which free fatty acids were extracted twice with petroleum ether, followed by evaporation to dryness under N_2 flow. Fatty acids were derivatized to its methyl ester derivative by adding 500 µL of methanolic HCl, incubating at 70 ºC for 1 h and evaporating under N_2 flow. Fatty acids derivatives were dissolved in hexane for GC/MS analysis under chemical ionization mode. Samples were injected at 250 ºC and the oven temperature was programmed as follows: 120 ºC for 1 min, then increased at 5 ºC/min to 220 ºC and held for 1 min. Detector was run in SIM recording ion abundance in the range of 269-279 m/z for palmitate and of 297-307 m/z for stearate. The retention times were 9.2 min for palmitate and 11.9 min for stearate.

Intracellular metabolic intermediates

Polar intracellular metabolites were extracted from liquid nitrogen-frozen cell cultured plates with the addition of 100% methanol and milli-Q H2O (1:1) and scraping on ice. Chloroform was added to cell lysates and tubes were placed in a shaker for vigorous agitation at 4 ºC for 30 min. Subsequently, samples were centrifuged at 4,000 rpm for 15 min and the upper aqueous phase was separated and evaporated to dryness under airflow at room temperature. TCA cycle intermediates and intracellular metabolites were derivatized by adding 50 µL of 2% (v/v) methoxyamine hydrochloride in pyridine to samples and shaking vigorously at 37 ºC for 90 min. Next, 30 µL of N-methyl-N-(tert-butyldimethylsilyl)trifluoroacetamide (MBTSTFA) + 1% tert-butyldimetheylchlorosilane (TBDMCS) were added and samples were incubated for 1 h at 55 ºC before GC/MS analysis under electron impact mode. Samples were injected at 270 ºC and the oven temperature was programmed as follows: 100 ºC for 3 min, then increased to 165 ºC at 10 ºC min^{-1}, then at 2.5 ºC/min to 225 ºC, at 25 ºC/min to 265 ºC and finally at 7.5 ºC/min to 300 ºC. The detector was run in SIM mode recording ion abundance in the range of 345 –356 m/z for C1-C5 α-ketoglutarate (RT: 24.6 min), 259 – 268 m/z for C1-C3 alanine (RT: 12.6 min), 417 – 428 m/z for C1-C4 aspartate (RT: 28.9 min), 458 – 469 m/z for C1-C6 citrate (RT: 37.7 min), 590 – 599 m/z for C1-C6 citrate (RT: 37.7 min), 329 – 338 m/z for C2-C5 glutamate (RT: 32.6 min), 431 – 442 m/z for C1-C5 glutamate (RT: 32.6 min), 260 – 269 m/z for C1-C3 lactate (RT: 11.8 min), 418 – 428 m/z for C1-C4 malate (RT: 27.6 min), 173 – 181 m/z for C1-C3 pyruvate (RT: 8.2 min).

Glycogen

Cells were washed twice with ice-cold PBS, scraped with 80 µL of 0.1 M NaOH and heated at 100 ºC for 15 min for protein denaturation. Then, samples were sonicated for 5 min using an ultrasonic bath (Branson 200 Ultrasonic Cleaner, Emerson Industrial Automation, St Louis, MO, USA). 5-10 µL of 1 µg/µL [U-^{13}C-D_7]-glucose were added as a recovery and internal standard to quantify the glucose released from glycogen. Cell extracts were neutralized with 0.5 M HCl and glycogen was digested by incubation with 1 U/mL α-amyloglucosidase in 0.4 M acetate buffer with gentle rocking for 20 h at 37 ºC. Both glucose released from glycogen and [U-^{13}C-D7]-glucose were isolated from homogenates using a tandem set of Dowex-1X8/Dowex-50WX8 ion-exchange columns, eluted with water. The glucose eluate was evaporated to dryness under airflow overnight. Glucose was converted to its glucose aldonitrile pentaacetate derivative after treatment for 30 min at 100 ºC with 100 µL of hydroxylamine hydrochloride in pyridine (2% v/v) and then with 75 µL of acetic anhydride for 1 h at 100 ºC. Excess reagent and solvent were removed by evaporation with N_2 flow, and the derivatized glucose was resuspended in ethyl acetate for GC/MS analysis under chemical ionization mode. Samples were injected at 250 ºC and the oven temperature was programmed as follows: 230 ºC for 2 min, then increased at 10 ºC/min to 260 ºC,

followed by a 25 ºC/min ramp to 270 ºC and held for 2 min. The detector was run in SIM, recording ion abundance of C1-C6 molecule in the range of 327-334 m/z for glucose and 339-345 m/z for the molecular ion (C1-C6) of the aldonitrile pentaacetate of the [U-^{13}C-D_7]-glucose used as a recovery and internal standard. The retention time was 3.7 min. Glucose from glycogen was normalized by cell number.

GC/MS data reduction

The ion clusters around specific m/z were monitored for each analyzed metabolite to determine the fractional distribution of ^{13}C. The peak areas for all ions in the cluster were extracted from raw data using MSD5975C Data Analysis (Agilent Technologies) or GCMS Postrun Analysis (Shimadzu Corporation) software. Each peak area is proportional to the fraction of ions with the same molecular weight. The value for each observed m/z is given by the experimental isotope incorporation, the presence of isotopes in heteroatoms, the presence of natural abundance of ^{13}C in the background and, when applicable, the ^{12}C isotope impurity in the ^{13}C-labeled precursor used as a tracer (in the case of glucose or glutamine). Also, derivatization reagents often contain isotopes (e.g. silicon, Si, isotopes) which contribute to the isotopologue distribution of the derivatized compound as well. Correction for all such contributions was conducted by regression analysis using an in-house developed algorithm. The algorithm used corrected all the previous detailed contributions over the observed spectral intensities of each ion cluster, and provided the isotopologue distribution in the analyzed metabolite due to incorporation of ^{13}C atoms from the tracer used as precursor. Results of the isotopologues in any of the ion clusters were reported as fractional enrichments or abundances, defined as the fraction of molecules having a certain number of isotope substitutions. Thus, they are designated as m0, m1, m2, etc. where the number indicates the number of labeled carbons (^{13}C) in the molecule corrected as described above. It is worth noting that the sum of fractional enrichments of all isotopologues of the ion clusters ($\sum_{i=0}^{i=n} m_i$, where n is the number of carbons in the molecule or fragment) is equal to 1 (or 100%), while the total ^{13}C enrichment is calculated as $\sum_{i=1}^{i=n} m_i$, or 1 (or 100%) minus m0.

Isotopic steady state and total ^{13}C enrichment

Ideally, assuming steady state, the distribution of isotopologues would only depend on the distribution of fluxes and the labeled and non-labeled status of the substrates used in the experiment. However, ^{13}C propagation from tracer precursors to products is a dynamic phenomenon. Initially, all product metabolites are unlabeled (m0). Progressively, these products are enriched in ^{13}C, with concomitant decrease in m0. Isotopic steady state (Selivanov et al., 2005) is quickly reached for small pools of metabolites but not necessarily for larger pools such as those of fatty acids, glycogen and culture medium metabolites. For these larger pools, m0 are oversized and may not decrease to the hypothetical value that should be reached at steady state. Accordingly, when isotopic steady state cannot be assumed, the measure of total ^{13}C enrichment ($\Sigma m = \sum_{i=1}^{i=n} m_i$) and the normalization of the isotopologue enrichments (m1, m2, m3, etc.) by Σm (m1/Σm, m2/Σm, m3/Σm, etc.) are used for comparisons.

^{13}C metabolic flux analysis and model description

We constructed a quantitative metabolic network model of central carbon metabolism and performed ^{13}C metabolic flux analysis by using the computer software INCA v1.5 (Young, 2014). We used as measured references experimental data including: 1) cellular uptake and production rates of glucose, lactate and all amino acids; 2) oxygen consumption rate; 3) glycogen accumulation rate; 4) protein synthesis rate and 5) two independent experiments of labeling propagation. The first labeling experiment measured by mass spectrometry ^{13}C propagation from 100% [1,2-$^{13}C_2$]-D-glucose to RNA-ribose, glycogen, palmitate, medium-glucose, medium-serine, medium-lactate, medium-alanine, medium-glutamine, medium-proline, cell-citrate, cell-α-ketoglutarate, cell-glutamate, cell-malate, cell-aspartate and cell-alanine. The second labeling experiment measured by mass spectrometry ^{13}C propagation from 100% [U-$^{13}C_5$]-L-glutamine to palmitate, medium-lactate, medium-alanine, medium-glutamine, medium-proline, cell-citrate, cell-α-ketoglutarate and cell-alanine.

By assuming isotopic steady state with INCA: 1) a parameter optimization process was applied, including the generation of statistical metrics used to assess the goodness of the fit; and 2) confidence intervals of the entire flux map were estimated to convey the uncertainties associated with all estimated parameters. The calculations of these confidence intervals were performed by selecting the parameter continuation method, which gradually vary each adjustable flux parameter, one at-a-time, from its optimal value while adjusting the remaining parameters to minimize the sum-of-squared residuals (SSR) (Antoniewicz et al., 2006). INCA is based on MATLAB; we used the MATLAB R2012b version.

The Table EV1 – 13C metabolic flux analysis contains: 1) model description, including model structure, reaction stoichiometry and atom transitions for ^{13}C propagation; 2) list of metabolites, including

abbreviations; 3) protocols used for mass spectrometry (MS) for each labeled metabolite; data generated by the program INCA, including 4) measured and predicted values for parameter optimization and statistical metrics used to assess goodness-of-fit and 5) predicted values for best fit obtained by parameter optimization, confidence intervals that convey the uncertainties associated with these estimated parameters and fold changes based on best fits. Also, two files, one per condition, were generated by INCA with the complete model, including the estimated flux map distributions. These files are available at doi.org/10.5281/zenodo.546717.

A complementary description of the objectives, procedures and assumptions used with our model are provided below:

1. The scope of the metabolic network covered central carbon metabolism: glycolysis, TCA cycle, PPP, glycogen metabolism, metabolism of uptake and oxidation of amino acid and fatty acid synthesis. Protein synthesis was included to balance the exchange and utilization of amino acids. The selection of the processes included in this metabolic network was based on databases of human metabolism such as BIGG database (Schellenberger et al., 2010) (including the human genome scale network reconstruction, *recon 1* (Duarte et al., 2007)), metacyc (Caspi et al., 2016), ExPASy (Artimo et al., 2012) and KEGG (Kanehisa et al., 2012). Generic (Nelson and Cox, 2008) and specific literature was used for proline (Liu et al., 2012; Liu and Phang, 2012; Phang et al., 2015; Phang et al., 2008), glycine and serine metabolisms (Dolfi et al., 2013; Fan et al., 2014; Jain et al., 2012; Tedeschi et al., 2013).
2. Rates for some reaction or transport processes were based on extracellular measurements and used as constraints in the fitting procedure. Thus, from the measured changes in media concentrations (see *"Estimation of metabolite consumption and production rates"*), the net uptake and release fluxes (rates) per cell of glucose (E1), lactate (E2), and all amino acids (E3-E22) were estimated from the experimental variation of concentration in media and the changes in the number of cells measured for 24 hours. Analogously, from the measured values of glycogen and protein per cell, we estimated the net flux of glycogen accumulation (E23) and the net flux of protein synthesis (E24) per cell and per glucose residue or amino acid, respectively. For proteins, an average molecular weight of 40,835 g/mol and an average length of 355 amino acids were assumed according to Dolfi *et al.* (Dolfi et al., 2013). Finally, measured rates of O_2 consumption for ATP production by mitochondrial respiration (E25) were also considered from OCR experiments (see "*Oxygen consumption rate (OCR) and extracellular acidification rate (ECAR)"*).
3. ATP and ADP were the only ribonucleoside phosphates considered in the model. For reactions involving other ribonucleoside phosphates, such as AMP, UTP, UDP, CTP and CDP, these were substituted by ATP or ADP, assuming the interconvertibility of ribonucleoside phosphates by adenylate kinase, nucleoside monophosphate kinases and nucleoside diphosphate kinases. Analogously, oxidized and reduced nicotinamide adenine dinucleotides (NAD and NADH, respectively) were considered in cytoplasm and mitochondria. Oxidized and reduced nicotinamide adenine dinucleotides phosphates (NADP and NADPH, respectively) were considered only in the cytoplasm, but not in mitochondria. In mitochondria, an equilibrium is assumed for all nicotinamide adenine dinucleotides forms involving the activities of nicotinamide nucleotide transhydrogenases (Hoek and Rydstrom, 1988) and the mitochondrial enzymes that can use as substrates both the phosphorylated and non-phosphorylated forms.
4. Inside the cell, a unique cell compartment is assumed for almost all species, since the experimental data of label propagation of Krebs cycle intermediaries (citrate, α-ketoglutarate, glutamate, malate and aspartate) are measured in cell pellets and is not possible to distinguish between cytosol and mitochondria. However, an exception was made for the oxidized and reduced forms of nicotinamide adenine dinucleotides (NAD and NADH, respectively), coenzyme A (CoA), acetyl-CoA and oxaloacetate, which are assumed to be in two possible compartments: cytosol or mitochondria. Having these metabolites in separate compartments allow to have enzymes that are functional in one or the other compartment, such as malate dehydrogenase, and allow for simulating the import of reducing equivalents from the cytosolic NADH into mitochondria by the malate-aspartate shuttle or the export of acetyl groups equivalents out of the mitochondria by the citrate-pyruvate/malate shuttle.
5. Extra reactions for ATP and NADPH utilization (R58 and R59, respectively) and for the recycling of mitochondrial acetyl-CoA (R60) were included to have appropriate balances of productions and consumptions. In contrast, no extra processes were added for cytoplasmic and mitochondrial NAD and NADH and for cytoplasmic acetyl-CoA and CoA, which are assumed to be well balanced with the specific reactions included in the model. The three reactions R58, R59 and R60 are not displayed in Figure 3.

6. Overall reactions are included for degradation of isoleucine, leucine, methionine, threonine, tryptophan, valine, cysteine, histidine and tyrosine. These and other reactions allowed to model the degradation of amino acids to mitochondrial substrates, including NADH.m, ACoA.m, succinate, fumarate, glutamate, pyruvate and acetyl-CoA and recycling of glutamate from α-ketoglutarate, which can be subsequently oxidized to α-ketoglutarate.
7. There are two reactions involved in oxygen consumption by mitochondrial respiratory chain, but a unique measurement of oxygen consumption (E25). A "virtual variable" pOCR was used to describe this process and included in reactions involving the entry of electrons through respiratory-chain-complex I (NADH dehydrogenase, R21) and respiratory-chain-complex II (succinate dehydrogenase, R20), such as at steady state the total oxygen consumption rate (E25) sum up those involving the entry of electrons through respiratory-chain-complex I and II: E25 = R21 + R20.
8. For the prediction of ^{13}C label enrichment in large pools, such as metabolites in the medium (medium-glucose, medium-serine, medium-alanine, medium-glutamine, medium-proline), glycogen, RNA (ribose) and palmitate, an isotopic steady state is never reached, and an excess of non-labeled isotopomers (m0) is expected. In order to correct for the excess of non-labeled isotopomers in these pools, fluxes were included to simulate the import of unlabeled metabolites. If appropriate for the balance of these large pools, an export flux was also included.

Intracellular metabolic intermediates quantification

Intracellular metabolic intermediates including pyruvate, alanine, malate, aspartate, α-ketoglutarate, glutamate and citrate were extracted and analyzed as above, with addition of 5 µL of norvaline (1 mg/mL) at each sample before scraping to minimize the differences associated with extraction efficiency. Chromatograms were integrated and areas were normalized by norvaline and cell number, and expressed as relative to control cells.

Polyamine quantification

Polyamines were extracted from liquid nitrogen-frozen cultured plates with addition of 500 µL of 10 mM acetic acid in methanol/Milli-Q water (1:1) and scraping on ice. Then, 5 µL of 200 mg/mL 1,6-diaminohexane (a total of 1 µg per sample) were added to each sample as an internal standard and cell extract were sonicated with a titanium probe (3 cycles, 5 sec per cycle, Tune 50, Output 30). The volume of each sample was measured and 20 µL were taken for protein determination. Samples were then centrifuged at 13,000 rpm at 4 ºC and the supernatant transferred to glass tubes for derivatization. First, pH was adjusted to 11-12 with 2 N NaOH. Then, 1 mL of diethyl ether and 50 µL of ethyl chloroformate were added and tubes were placed in a shaker for vigorous agitation at 4 ºC for 20 min. Subsequently, samples were centrifuged at 2,500 g for 5 min and the organic phase was collected and evaporated to dryness under N_2 flow. Next, 20 µL of pentaflyoropropylic anhydride and 100 µL of ethyl acetate were added and samples were incubated at 50 ºC for 30 min. Finally, samples were dried under N_2 flow and resuspended with ethyl acetate for GC/MS analysis under electron impact mode. Samples were injected at 260 ºC and the oven temperature was programmed as follows: 140 ºC, then increased to 210 ºC at 8 ºC/min and held for 2 min and finally at 20 ºC/min to 320 ºC and held for 3.75 min. The retention times were 7.99 min for putrescine, 14.95 min for spermidine and 10.63 for 1,6-diaminohexane.

Oxygen consumption rate (OCR) and extracellular acidification rate (ECAR)

Oxygen consumption rate (OCR) and extracellular acidification rate (ECAR) were determined using a XF24 Extracellular Flux Analyzer (Seahorse Bioscience, North Billerica, MA, USA). Cells were collected and reseeded at a concentration of 6×10^4 cells per well in 100 µL of complete medium in a XF24-well microplate (Seahorse Bioscience). One hundred and fifty µL of complete medium were added to the wells 4 h after seeding (once the cells were attached) and plates were incubated at 37 ºC and 5% CO_2. After 24 h, 150 µL of medium was removed from each well (to prevent cell exposure to air and potentially drying out) and cells were rinsed with 1 mL of warm XF assay medium (non-buffered, pH 7.4, Seahorse Bioscience) supplemented exclusively with the carbon source indicated in each case (glucose, glutamine or neither glucose nor glutamine). Finally, 400 µL of XF assay medium were added to each well and plates were incubated at 37 ºC for 1 h without CO_2. Previously, the sensor cartridge was incubated overnight with 1 mL of PBS per well at 37 ºC. Oxygen and proton measurements were carried out over 105 minutes divided into five periods, following the programmed protocol. Within the first period, basal oxygen consumption rate and basal extracellular acidification rate were determined. Once the measure was complete, the cells from each well were counted to normalize the OCR and ECAR readings.

Mito Stress test

Mitochondrial function was analyzed by sequential injections of oligomycin (ATP synthase inhibitor), carbonyl cyanide 4-(trifluoromethoxy) phenylhydrazone (FCCP, mitochondrial uncoupler) and rotenone and antimycin A (mitochondrial complex I and III inhibitors, respectively). For the different experiments using this test, four types of XF assay media were used: minimal media without glucose and glutamine, media supplemented with 10 mM glucose, media supplemented with 2 mM glutamine and complete media supplemented with 10 mM glucose and 2 mM glutamine.

Other data analysis and statistical methods

Experiments were carried out at least in triplicate and repeated two or three times. Samples were selected randomly for drug treatments. To evaluate the effects of combined drug treatments, the Chou–Talalay multiple drug-effect analysis (Chou and Talalay, 1984) was used with the CompuSyn software (ComboSyn, Inc., Paramus, NJ, USA). Combined drug treatment interactions were quantified by determining the Combination Index (CI), where $CI<1$, $CI=1$, and $CI>1$ indicate synergism, additivity, and antagonism, respectively. Statistical analyses were conducted using Statgraphics statistical package (Statgraphics Centurion XVI, StatPoint technologies Inc., Warrenton, VA, USA). Fisher's least significant difference (LSD) test was used to identify the groups that significantly differed from each other. Shapiro-Wilk test was used to assess normal distribution of experimental data. Outliers were identified by Dixon's Q-test and homogeneity of variances was assessed by Levene's test. Control and treatment measurements were compared using Kruskal-Wallis, ANOVA and two-tailed independent sample Student's t tests. All data are expressed as mean ± standard deviation (SD). Differences were considered to be significant at $p < 0.05$ (*), $p < 0.01$ (**) and $p < 0.001$ (***). Not-significant differences ($p > 0.05$) are indicated in some cases as n.s.

3. Appendix References

Anastassiadis, T., Deacon, S. W., Devarajan, K., Ma, H., and Peterson, J. R. (2011). Comprehensive assay of kinase catalytic activity reveals features of kinase inhibitor selectivity. Nat Biotechnol *29*, 1039-1045.

Antoniewicz, M. R., Kelleher, J. K., and Stephanopoulos, G. (2006). Determination of confidence intervals of metabolic fluxes estimated from stable isotope measurements. Metab Eng *8*, 324-337.

Artimo, P., Jonnalagedda, M., Arnold, K., Baratin, D., Csardi, G., de Castro, E., Duvaud, S., Flegel, V., Fortier, A., Gasteiger, E*., et al.* (2012). ExPASy: SIB bioinformatics resource portal. Nucleic Acids Res *40*, W597-603.

Benjamini, Y., and Hochberg, Y. (1995). Controlling the False Discovery Rate: A Practical and Powerful Approach to Multiple Testing. J Roy Statist Soc Ser B (Methodological) *57*, 289-300.

Brady, D. C., Crowe, M. S., Turski, M. L., Hobbs, G. A., Yao, X., Chaikuad, A., Knapp, S., Xiao, K., Campbell, S. L., Thiele, D. J., and Counter, C. M. (2014). Copper is required for oncogenic BRAF signalling and tumorigenesis. Nature *509*, 492-496.

Caspi, R., Billington, R., Ferrer, L., Foerster, H., Fulcher, C. A., Keseler, I. M., Kothari, A., Krummenacker, M., Latendresse, M., Mueller, L. A*., et al.* (2016). The MetaCyc database of metabolic pathways and enzymes and the BioCyc collection of pathway/genome databases. Nucleic Acids Res *44*, D471-480.

Chou, T. C., and Talalay, P. (1984). Quantitative analysis of dose-effect relationships: the combined effects of multiple drugs or enzyme inhibitors. Adv Enzyme Regul *22*, 27-55.

Dolfi, S. C., Chan, L. L., Qiu, J., Tedeschi, P. M., Bertino, J. R., Hirshfield, K. M., Oltvai, Z. N., and Vazquez, A. (2013). The metabolic demands of cancer cells are coupled to their size and protein synthesis rates. Cancer Metab *1*, 20.

Duarte, N. C., Becker, S. A., Jamshidi, N., Thiele, I., Mo, M. L., Vo, T. D., Srivas, R., and Palsson, B. O. (2007). Global reconstruction of the human metabolic network based on genomic and bibliomic data. Proceedings of the National Academy of Sciences of the United States of America *104*, 1777-1782.

Edgar, R., Domrachev, M., and Lash, A. E. (2002). Gene Expression Omnibus: NCBI gene expression and hybridization array data repository. Nucleic Acids Res *30*, 207-210.

Fan, J., Ye, J., Kamphorst, J. J., Shlomi, T., Thompson, C. B., and Rabinowitz, J. D. (2014). Quantitative flux analysis reveals folate-dependent NADPH production. Nature *510*, 298-302.

Hoek, J. B., and Rydstrom, J. (1988). Physiological roles of nicotinamide nucleotide transhydrogenase. The Biochemical journal *254*, 1-10.

Irizarry, R. A., Bolstad, B. M., Collin, F., Cope, L. M., Hobbs, B., and Speed, T. P. (2003). Summaries of Affymetrix GeneChip probe level data. Nucleic Acids Res *31*, e15.

Jain, M., Nilsson, R., Sharma, S., Madhusudhan, N., Kitami, T., Souza, A. L., Kafri, R., Kirschner, M. W., Clish, C. B., and Mootha, V. K. (2012). Metabolite profiling identifies a key role for glycine in rapid cancer cell proliferation. Science (New York, NY *336*, 1040-1044.

Kanehisa, M., Goto, S., Sato, Y., Furumichi, M., and Tanabe, M. (2012). KEGG for integration and interpretation of large-scale molecular data sets. Nucleic Acids Res *40*, D109-114.

Liu, W., Le, A., Hancock, C., Lane, A. N., Dang, C. V., Fan, T. W. M., and Phang, J. M. (2012). Reprogramming of proline and glutamine metabolism contributes to the proliferative and metabolic responses regulated by oncogenic transcription factor c-MYC. Proc Natl Acad Sci U S A *109*, 8983-8988.

Liu, W., and Phang, J. M. (2012). Proline dehydrogenase (oxidase) in cancer. Biofactors *38*, 398-406.

Morgenstern, J. P., and Land, H. (1990). Advanced mammalian gene transfer: high titre retroviral vectors with multiple drug selection markers and a complementary helper-free packaging cell line. Nucleic Acids Res *18*, 3587-3596.

Mosmann, T. (1983). Rapid colorimetric assay for cellular growth and survival: application to proliferation and cytotoxicity assays. J Immunol Methods *65*, 55-63.

Moyer, M. P., Manzano, L. A., Merriman, R. L., Stauffer, J. S., and Tanzer, L. R. (1996). NCM460, a normal human colon mucosal epithelial cell line. In Vitro Cell Dev Biol Anim *32*, 315-317.

Nelson, D. L., and Cox, M. M. (2008). Lehninger Principles of Biochemistry, fifth edn (New York: W.H. Freeman and Company).

Perkins, D. N., Pappin, D. J., Creasy, D. M., and Cottrell, J. S. (1999). Probability-based protein identification by searching sequence databases using mass spectrometry data. Electrophoresis *20*, 3551-3567.

Phang, J. M., Liu, W., Hancock, C. N., and Fischer, J. W. (2015). Proline metabolism and cancer. Curr Opin Clin Nutr Metab Care *18*, 71-77.

Phang, J. M., Pandhare, J., Zabirnyk, O., and Liu, Y. (2008). PPARgamma and Proline Oxidase in Cancer. PPAR Res *2008*, 542694.

Rabinovitch, P. S. (1994). DNA content histogram and cell-cycle analysis. Methods Cell Biol *41*, 263-296.
Schellenberger, J., Park, J. O., Conrad, T. M., and Palsson, B. O. (2010). BiGG: a Biochemical Genetic and Genomic knowledgebase of large scale metabolic reconstructions. BMC Bioinformatics *11*, 213.
Selivanov, V. A., Meshalkina, L. E., Solovjeva, O. N., Kuchel, P. W., Ramos-Montoya, A., Kochetov, G. A., Lee, P. W., and Cascante, M. (2005). Rapid simulation and analysis of isotopomer distributions using constraints based on enzyme mechanisms: an example from HT29 cancer cells. Bioinformatics *21*, 3558-3564.
Sheikh, K., Forster, J., and Nielsen, L. K. (2005). Modeling hybridoma cell metabolism using a generic genome-scale metabolic model of Mus musculus. Biotechnol Prog *21*, 112-121.
Smyth, G. K. (2004). Linear models and empirical bayes methods for assessing differential expression in microarray experiments. Stat Appl Genet Mol Biol *3*, Article3.
Subramanian, A., Tamayo, P., Mootha, V. K., Mukherjee, S., Ebert, B. L., Gillette, M. A., Paulovich, A., Pomeroy, S. L., Golub, T. R., Lander, E. S., and Mesirov, J. P. (2005). Gene set enrichment analysis: a knowledge-based approach for interpreting genome-wide expression profiles. Proceedings of the National Academy of Sciences of the United States of America *102*, 15545-15550.
Tedeschi, P. M., Markert, E. K., Gounder, M., Lin, H., Dvorzhinski, D., Dolfi, S. C., Chan, L. L. Y., Qiu, J., DiPaola, R. S., Hirshfield, K. M., *et al.* (2013). Contribution of serine, folate and glycine metabolism to the ATP, NADPH and purine requirements of cancer cells. Cell Death Dis *4*, e877.
Wilson, C. L., and Miller, C. J. (2005). Simpleaffy: a BioConductor package for Affymetrix Quality Control and data analysis. Bioinformatics *21*, 3683-3685.
Wisniewski, J. R., Zougman, A., Nagaraj, N., and Mann, M. (2009). Universal sample preparation method for proteome analysis. Nat Methods *6*, 359-362.
Young, J. D. (2014). INCA: a computational platform for isotopically non-stationary metabolic flux analysis. Bioinformatics *30*, 1333-1335.
