# Supplementary material for: De novo MYC addiction as an adaptive response of cancer cells to CDK4/6 inhibition
Source: Mol Syst Biol. 2017 Oct 4;13(10):940. doi: 10.15252/msb.20167321 (PMC5658703; doi:10.15252/msb.20167321)
Supplement: Supplementary file 2 — Expanded View Figures PDF [file MSB-13-940-s002.pdf]

Expanded View Figures

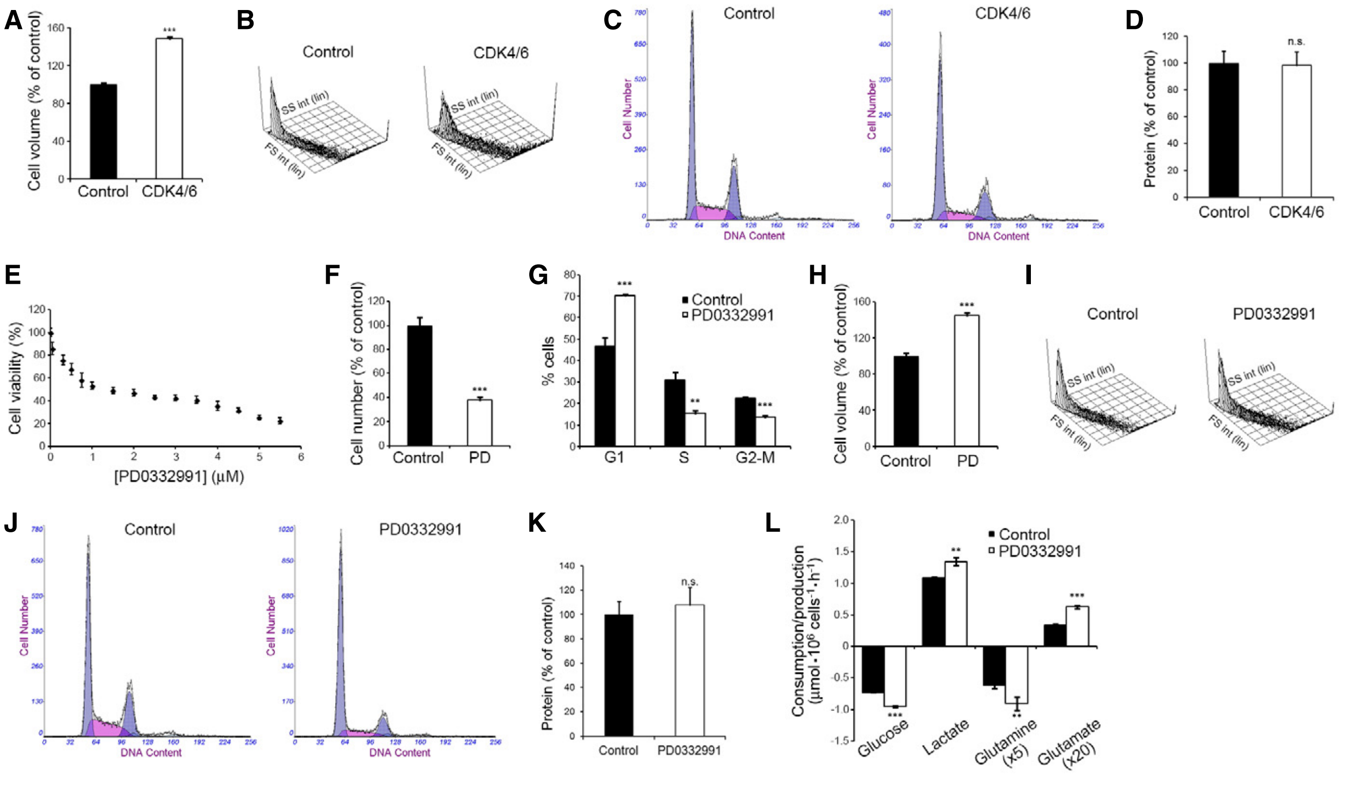

Figure EV1. Effects of CDK4/6 inhibition on the growth and metabolic fluxes of HCT116 cells.

- A Cell volume of CDK4/6-kd and control cells measured 96 h after transfection. Results are represented as percentage of volume relative to control cells.
- B Forward Scatter (FS) versus Side Scatter (SS) plot of CDK4/6-kd and control cells.
- C DNA content analyses of CDK4/6-kd and control cells determined by flow cytometry.
- D Total protein content of CDK4/6-kd and control cells. Results are represented as percentage of protein relative to control cells.
- E Viability curve of HCT116 cells in response to PD0332991. Exponentially growing cells were treated with the indicated concentrations of PD0332991 for 96 h, and cell proliferation was determined by Hoechst staining. Results are represented as percentage of proliferation relative to untreated cells. IC50 was estimated at 1.7 μM. Values correspond to averages of three independent experiments (n = 6 per experiment).
- F Effect of treatment with PD0332991 (2 μM) on cell proliferation, determined by flow cytometry combining direct cell counting and PI staining.
- G Cell cycle analysis of PD0332991-treated and control cells.
- H Cell volume of PD0332991-treated and control cells. Results are represented as percentage of volume relative to control cells.
- I Forward Scatter (FS) versus Side Scatter (SS) plot of PD0332991-treated and control cells.
- J DNA content analyses of PD0332991-treated and control cells determined by flow cytometry.
- K Total protein content of PD0332991-treated and control cells. Results are represented as percentage of protein relative to control cells.
- L Comparative extracellular metabolic fluxes for PD0332991-treated and control cells. Glucose and glutamine consumption and lactate and glutamate production rates were obtained after 24 h of incubation with fresh media and normalized to cell number.

Data information: Data are shown as mean ± SD. Statistically significant differences between CDK4/6-inhibited and control cells were determined by two-tailed independent sample Student's t-tests and are indicated at $P < 0.01$ (**) and $P < 0.001$ (***). n.s., statistically non-significant.

© 2017 The Authors

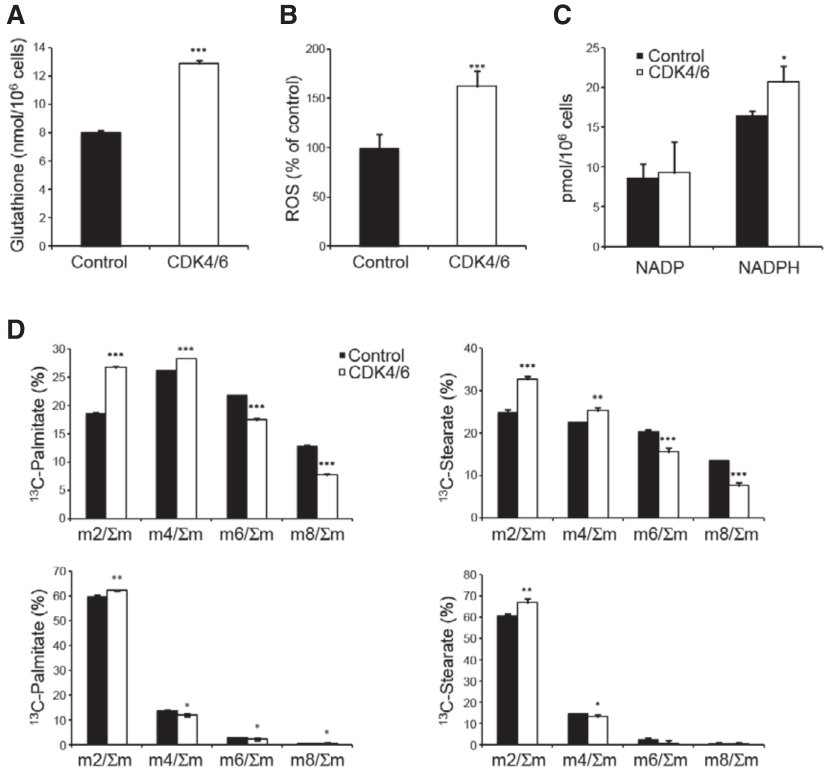

Figure EV2. Intracellular glutathione, ROS, NADPH levels and fatty acid synthesis in CDK4/6-kd and control cells.

- A Total intracellular glutathione content normalized to cell number.
- B Intracellular ROS levels determined by flow cytometry. Data are expressed as percentages of mean fluorescent intensity (MnX) relative to control cells.
- C NADP and NADPH levels quantified by a colorimetric assay using the NADP/NADPH Quantification Kit (MAK038, Sigma-Aldrich) and normalized to cell number.
- D Dynamic accumulation of isotopologues in palmitate and stearate after 24 h incubation with 10 mM [1,2-$^{13}C_2$]-glucose (top) or 2 mM [U-^{13}C]-glutamine (bottom), suggesting an impaired fatty acid synthesis in CDK4/6-kd cells.

Data information: CDK4/6, CDK4/6-kd cells; Control, non-targeting siRNA-transfected cells. Bars correspond to mean ± SD (n = 3). Statistically significant differences between CDK4/6-inhibited and control cells were determined by two-tailed independent sample Student's t-tests and are indicated as $P < 0.05$ (*), $P < 0.01$ (**), and $P < 0.001$ (***).

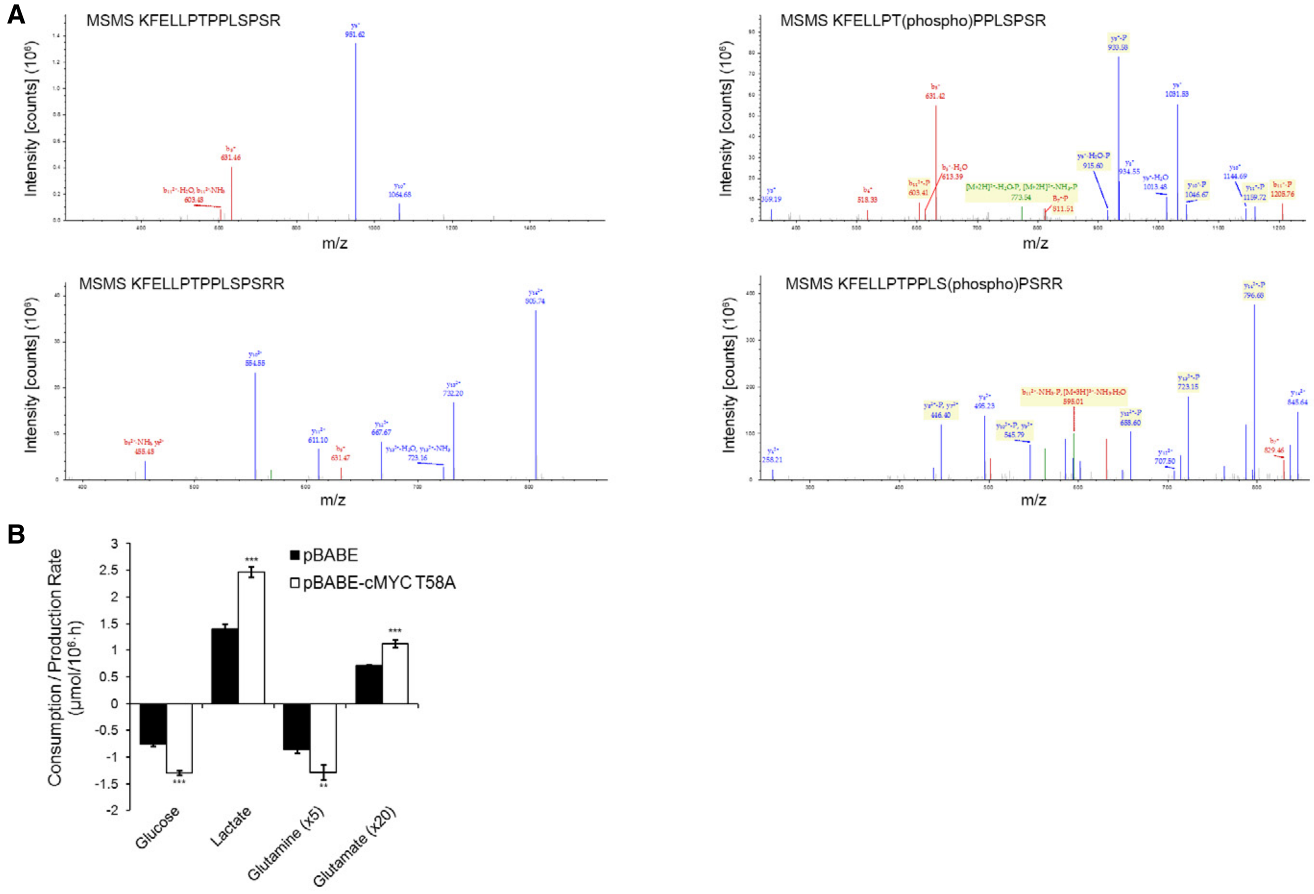

Figure EV3. CDK4/6 directly phosphorylates c-MYC at T58 and S62.

A Mass spectrometry analysis of the two c-MYC peptides that were found to be phosphorylated by CDK4/6-Cyclin D1 complexes. Kinase assays were performed with full-length recombinant human c-MYC adding CDK4-Cyclin D1 or CDK6-Cyclin D1 complexes and using (right) or not using (left) unlabeled ATP followed by a mass spectrometry phosphosite profiling assay. Samples from the kinase assay were digested with trypsin and analyzed with LC-MS/MS. Data were blindly assessed.

B Glucose and glutamine consumption and lactate and glutamate production rates in HCT116 cells expressing a T58A MYC mutant. Media were collected after 24 h of incubation with fresh media and results are normalized to cell number.

Data information: Bars correspond to mean ± SD (n = 3). Statistically significant differences between T58A MYC mutant-expressing and control cells were determined by two-tailed independent sample Student's t-tests and are indicated as $P < 0.01$ (**), and $P < 0.001$ (***).

© 2017 The Authors

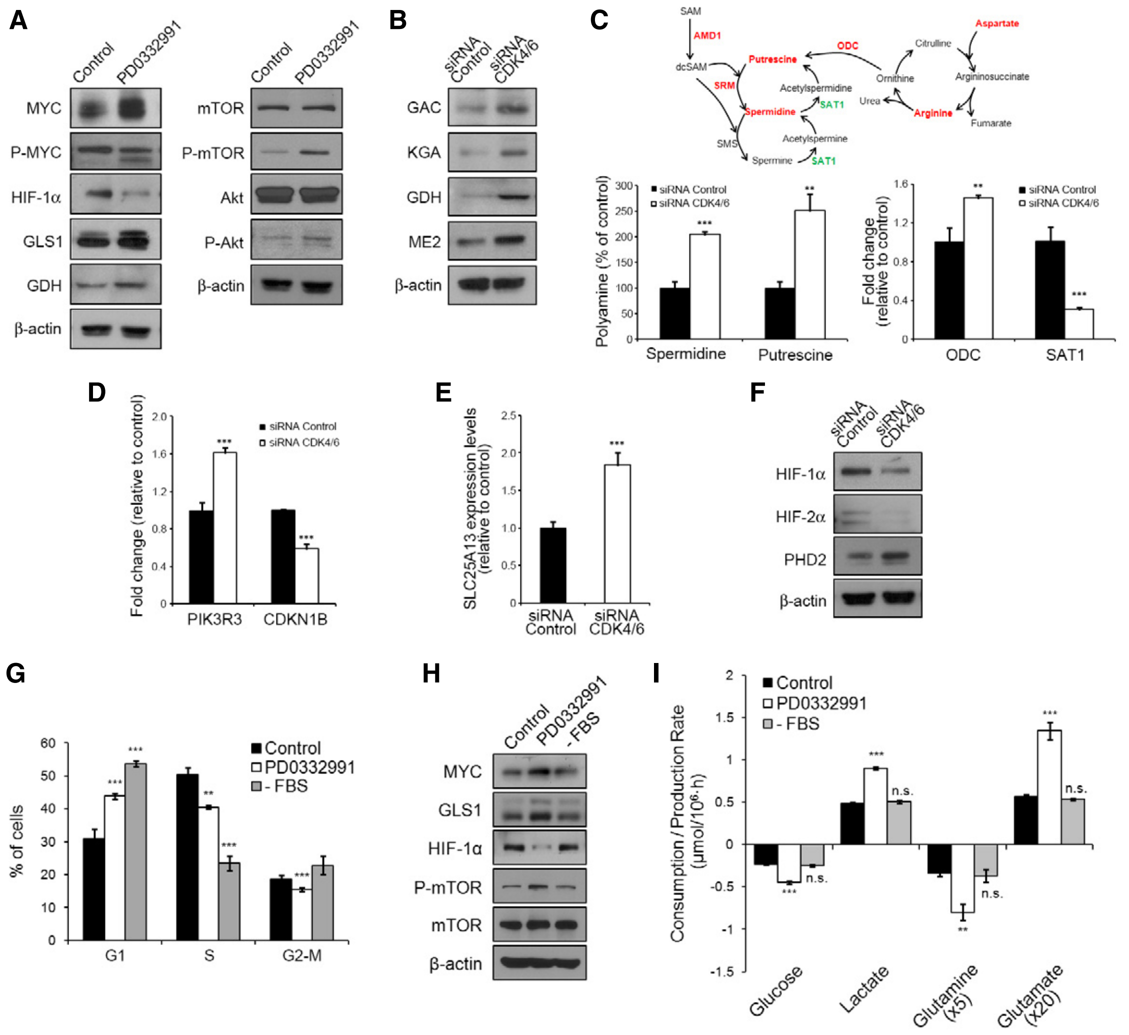

Figure EV4. Effects of CDK4/6 inhibition on signaling pathways.

A Western blotting analysis of total protein fractions of CDK4/6-inhibited and control cells. Protein extracts were obtained after incubating HCT116 cells with PD0332991 (2 μM) or vehicle for 96 h.

B Western blotting analysis of total protein fractions of CDK4/6-kd and control cells.

C Polyamine metabolism in CDK4/6-kd and control cells. Top, schematic representation of the polyamine metabolism and the urea cycle. The metabolites and enzymes which were found to be increased are depicted in red and those found to be downregulated in green. Bottom left, quantification of spermidine and putrescine polyamines by GC/MS. Results are shown as percentage of polyamine metabolite relative to control cells. Bottom right, qRT–PCR measures of *ODC* and *SAT1* gene expression. Data are normalized to cyclophilin A and expressed as fold change of mRNA relative to non-targeting siRNA-treated cells. Altogether, polyamine accumulation and changes in expression of enzymes involved in polyamine metabolism suggest an increase in polyamine synthesis in CDK4/6-kd cells.

D *PIK3R3* and *CDKN1B* gene expression levels determined by qRT–PCR in CDK4/6-kd and control cells 96 h after transfection. Results are normalized to cyclophilin A and expressed as fold change of mRNA relative to expression of non-targeting siRNA-treated cells.

E *SLC25A13* gene expression levels determined by qRT–PCR. Results are normalized to cyclophilin A and expressed as fold change of mRNA relative to expression of non-targeting siRNA-treated cells.

F HIF-1α, HIF-2α, and PHD2 protein levels were determined by Western blotting in CDK4/6-kd and control cells.

G Cell cycle distribution of NCM460 cells after PD0332991 treatment or serum deprivation.

H Western blotting analysis of total protein fractions of control, PD0332991-treated and serum-deprived NCM460 cells.

I Comparative extracellular metabolic fluxes for control, PD0332991-treated, and serum-starved NCM460 cells. Glucose and glutamine consumption and lactate and glutamate production rates were obtained after 24 h of incubation with fresh media and normalized to cell number.

Data information: Bars represent mean ± SD (n = 3). All experiments were performed 96 h after siRNA transfection or PD0332991 treatment, as specified in each case. β-actin was employed as a protein loading control in all Western blotting experiments. Statistically significant differences between CDK4/6-kd and control cells or serum-starved and control cells were determined by two-tailed independent sample Student's t-tests and are indicated as $P < 0.01$ (**) and $P < 0.001$ (***). n.s., statistically non-significant.

Source data are available online for this figure.

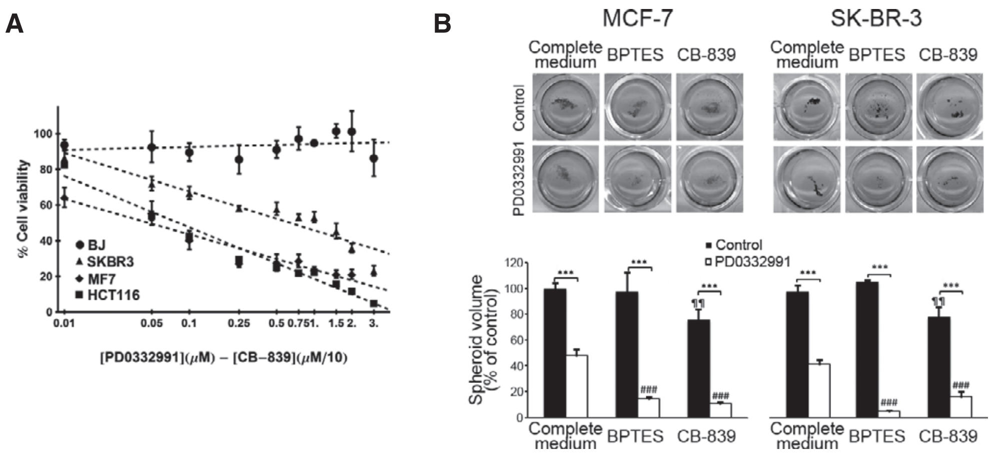

Figure EV5. CDK4/6 and glutaminase inhibition as a combined therapy.

A The combination of PD0332991 with the GLS1 inhibitor CB-839 is not toxic to BJ fibroblasts. BJ normal human fibroblasts, HCT116 colorectal cancer cells, and MCF-7 and SK-BR-3 breast cancer cells were cultured at the indicated concentrations of inhibitors for 96 h, and cell proliferation was determined by Hoechst staining. Results are shown as percentage of proliferation relative to untreated cells (mean ± SD of $n = 6$).

B Effects on spheroid growth of MCF-7 and SK-BR-3 breast cancer cells by CDK4/6 and GLS1 inhibitors. Cells were grown in low-attachment plates and incubated with the indicated combinations of PD0332991 (2 μM) with BPTES (10 μM) or CB-839 (10 μM) for 10 days. Top, representative images of spheroids after staining with MTT. Bottom, quantifications of spheroid volume (mean ± SD of $n = 4$), represented as percentages of total spheroid volume relative to untreated cells. Spheroids were scored by image acquisition and through spheroid area and volume quantification with ImageJ software. Significance was determined by Kruskal-Wallis and two-tailed independent sample Student's *t*-tests. Statistically significant differences between CDK4/6-inhibited and control cells were indicated at $P < 0.001$ (***), while differences between treatment (BPTES or CB-839) and the corresponding control (PD0332991-treated cells or untreated cells in complete medium) were shown at $P < 0.001$ (###) for CDK4/6-inhibited cells and at $P < 0.01$ (¶¶) for control cells.

© 2017 The Authors
