# Supplementary material for: De novo MYC addiction as an adaptive response of cancer cells to CDK4/6 inhibition
Source: Mol Syst Biol. 2017 Oct 4;13(10):940. doi: 10.15252/msb.20167321 (PMC5658703; doi:10.15252/msb.20167321)
Supplement: Supplementary file 6 — Table EV4 [file MSB-13-940-s006.docx]

**Table EV4. Synergistic antiproliferative effects of combined treatments.** Cells were treated for 96 h at the indicated concentrations (µM) of inhibitors at the shown constant ratios. The CI results obtained with CompuSyn software revealed a synergy (CI<1) in the antiproliferative effects of the combined treatments at each dose combination tested.

| **HCT116, 1:4 ratio** | | | | **HCT116, 10:1 ratio** | | | | |
| --- | --- | --- | --- | --- | --- | --- | --- | --- |
| **PD0332991 (µM)** | **BPTES** | **Viability (%)** | **CI** | **PD0332991 (µM)** | | **CB-839** | **Viability (%) (%)** | **CI value** |
| 0.5 | 2 | 40.3 ± 3.9 | 0.195 | 0.01 | | 0.001 | 82.7 ± 1.1 | 0.112 |
| 1 | 4 | 31.3 ± 2.5 | 0.227 | 0.05 | | 0.005 | 54.1 ± 2.5 | 0.09 |
| 1.5 | 6 | 17.7 ± 2.3 | 0.122 | 0.1 | | 0.01 | 42.8 ± 0.7 | 0.097 |
| 2 | 8 | 16.5 ± 0.9 | 0.146 | 0.25 | | 0.025 | 29.9 ± 0.2 | 0.113 |
| 2.5 | 10 | 11.8 ± 1.9 | 0.107 | 0.5 | | 0.05 | 25.1 ± 0.6 | 0.164 |
| 3 | 12 | 9.2 ± 1.7 | 0.088 | 0.75 | | 0.075 | 22.2 ± 1.0 | 0.197 |
| 3.5 | 14 | 7.9 ± 1.0 | 0.082 | 1 | | 0.1 | 22.7 ± 1.6 | 0.274 |
| 4 | 16 | 5.1 ± 1.3 | 0.049 | 1.5 | | 0.15 | 16.0 ± 0.6 | 0.214 |
| 4.5 | 18 | 5.0 ± 1.7 | 0.055 | 2 | | 0.2 | 12.0 ± 0.1 | 0.194 |
| 5 | 20 | 5.0 ± 0.7 | 0.060 | 3 | | 0.3 | 5.1 ± 0.1 | 0.820 |
| **MCF-7, 1:4 ratio** | | | | **MCF-7, 10:1 ratio** | | | | |
| 0.05 | 0.2 | 63.0 ± 4.6 | 0.163 | 0.01 | 0.001 | | 64.3 ± 5.6 | 0.036 |
| 0.1 | 0.4 | 56.8 ± 0.8 | 0.196 | 0.05 | 0.005 | | 55.5 ± 6.7 | 0.069 |
| 0.25 | 1 | 38.5 ± 2.6 | 0.13 | 0.1 | 0.01 | | 40.3 ± 5.3 | 0.03 |
| 0.5 | 2 | 31.1 ± 2.3 | 0.161 | 0.25 | 0.025 | | 27.4 ± 2.2 | 0.021 |
| 0.75 | 3 | 29.8 ± 2.6 | 0.222 | 0.5 | 0.05 | | 27.7 ± 4.8 | 0.045 |
| 1 | 4 | 28.7 ± 2.4 | 0.277 | 0.75 | 0.075 | | 28.8 ± 3.6 | 0.072 |
| 1.5 | 6 | 27.1 ± 2.0 | 0.377 | 1 | 0.1 | | 23.4 ± 2.1 | 0.055 |
| 2 | 8 | 24.9 ± 2.6 | 0.438 | 1.5 | 0.15 | | 21.3 ± 2.1 | 0.066 |
| 3 | 12 | 27.0 ± 1.9 | 0.636 | 2 | 0.2 | | 21.2 ± 2.7 | 0.087 |
| **SK-BR-3, 1:4 ratio** | | | | **SK-BR-3, 10:1 ratio** | | | | |
| 0.5 | 2 | 67.4 ± 2.8 | 0.222 | 0.01 | 0.001 | | 86.7 ± 3.9 | 0.07 |
| 1 | 4 | 59.1 ± 2.8 | 0.223 | 0.05 | 0.005 | | 72.1 ± 4.0 | 0.049 |
| 1.5 | 6 | 52.3 ± 1.3 | 0.197 | 0.1 | 0.01 | | 66.9 ± 3.5 | 0.059 |
| 2 | 8 | 44.8 ± 2.1 | 0.145 | 0.25 | 0.025 | | 58.5 ± 1.2 | 0.07 |
| 2.5 | 10 | 39.0 ± 0.1 | 0.112 | 0.5 | 0.05 | | 57.9 ± 3.6 | 0.133 |
| 3 | 12 | 38.7 ± 1.9 | 0.138 | 0.75 | 0.074 | | 53.2 ± 1.7 | 0.136 |
| 4 | 16 | 38.0 ± 1.0 | 0.169 | 1 | 0.1 | | 53.2 ± 3.0 | 0.18 |
| 5 | 20 | 34.7 ± 1.7 | 0.16 | 1.5 | 0.15 | | 45.4 ± 4.3 | 0.142 |
| 6 | 24 | 26.9 ± 0.9 | 0.094 | 2 | 0.2 | | 35.9 ± 2.6 | 0.085 |
| 7 | 28 | 23.4 ± 1.4 | 0.077 | 3 | 0.3 | | 23.3 ± 2.7 | 0.036 |

| **HCT116, 5:1 ratio** | | | | **HCT116, 1:1 ratio** | | | |
| --- | --- | --- | --- | --- | --- | --- | --- |
| **PD0332991 (µM)** | **Rapamycin** | **Viability (%)** | **CI** | **PD0332991 (µM)** | **LY294002** | **Viability (%) (%)** | **CI value** |
| 0.01 | 0.002 | 53.9 ± 3.7 | 0.005 | 0.05 | 0.05 | 73.8 ± 1.7 | 0.215 |
| 0.03 | 0.006 | 36.3 ± 3.0 | 0.009 | 0.1 | 0.1 | 69.7 ± 3.0 | 023 |
| 0.05 | 0.01 | 31.9 ± 4.9 | 0.011 | 0.5 | 0.5 | 49.2 ± 3.7 | 0.357 |
| 0.15 | 0.03 | 27.1 ± 1.4 | 0.022 | 1 | 1 | 39.3 ± 1.9 | 0.546 |
| 0.25 | 0.05 | 21.2 ± 2.9 | 0.022 | 1.5 | 1.5 | 25.1 ± 0.6 | 0.637 |
| 0.4 | 0.08 | 21.2 ± 2.4 | 0.035 | 2 | 2 | 37.5 ± 3.0 | 0.71 |
| 0.5 | 0.1 | 21.2 ± 0.9 | 0.044 | 2.5 | 2.5 | 28.6 ± 2.0 | 0.581 |
| 1 | 0.2 | 17.1 ± 1.7 | 0.057 | 3 | 3 | 24.4 ± 2.0 | 0.542 |
| 2 | 0.4 | 11.8 ± 1.7 | 0.056 | 4 | 4 | 17.3 ± 1.8 | 0.451 |
| 3 | 0.6 | 9.9 ± 1.1 | 0.061 | 4.5 | 4.5 | 14.1 ± 0.8 | 0.399 |
| **MCF-7, 5:1 ratio** | | | | **MCF-7, 1:1 ratio** | | | |
| 0.01 | 0.002 | 45.0 ± 6.2 | 0.013 | 0.05 | 0.05 | 52.6 ± 4.2 | 0.056 |
| 0.03 | 0.006 | 31.1 ± 3.2 | 0.004 | 0.1 | 0.1 | 46.8 ± 3.7 | 0.064 |
| 0.05 | 0.01 | 29.1 ± 0.3 | 0.005 | 0.5 | 0.5 | 39.6 ± 2.3 | 0.164 |
| 0.15 | 0.03 | 21.5 ± 1.7 | 0.004 | 1 | 1 | 29.0 ± 3.1 | 0.124 |
| 0.25 | 0.05 | 20.7 ± 1.2 | 0.006 | 1.5 | 1.5 | 28.0 ± 3.2 | 0.169 |
| 0.4 | 0.08 | 21.3 ± 3.0 | 0.01 | 2 | 2 | 23.9 ± 3.1 | 0.153 |
| 0.5 | 0.1 | 20.9 ± 1.7 | 0.012 | 2.5 | 2.5 | 23.7 ± 3.3 | 0.188 |
| 1 | 0.2 | 22.4 ± 0.9 | 0.031 | 3 | 3 | 21.0 ± 3.6 | 0.174 |
| 2 | 0.4 | 22.2 ± 1.3 | 0.006 | 4 | 4 | 14.6 ± 2.1 | 0.118 |
| 3 | 0.6 | 15.6 ± 3.2 | 0.025 | 4.5 | 4.5 | 12.3 ± 1.3 | 0.103 |
| **SK-BR-3, 5:1 ratio** | | | | **SK-BR-3, 1:1 ratio** | | | |
| 0.5 | 0.002 | 46.5 ± 7.3 | 0.064 | 0.05 | 0.05 | 73.8 ± 1.8 | 0.078 |
| 1 | 0.006 | 35.3 ± 4.0 | 0.004 | 0.1 | 0.1 | 66.0 ± 2.0 | 0.081 |
| 1.5 | 0.01 | 31.1 ± 4.0 | 0.002 | 0.5 | 0.5 | 55.0 ± 2.1 | 0.183 |
| 2 | 0.03 | 28.2 ± 1.6 | 0.004 | 1 | 1 | 51.1 ± 2.4 | 0.281 |
| 2.5 | 0.05 | 24.9 ± 4.4 | 0.004 | 1.5 | 1.5 | 42.4 ± 1.4 | 0.242 |
| 3 | 0.08 | 25.1 ± 1.4 | 0.006 | 2 | 2 | 35.9 ± 1.2 | 0.214 |
| 4 | 0.1 | 25.6 ± 0.1 | 0.008 | 2.5 | 2.5 | 32.8 ± 1.0 | 0.218 |
| 5 | 0.2 | 23.5 ± 1.9 | 0013 | 3 | 3 | 24.2 ± 3.1 | 0.143 |
| 6 | 0.4 | 23.8 ± 4.5 | 0.026 | 4 | 4 | 16.7 ± 6.5 | 0.101 |
| 7 | 0.6 | 20.0 ± 5.9 | 0.024 | 4.5 | 4.5 | 15.6 ± 6.3 | 0.103 |
|  |  | | | | | | |
