# Supplementary figures and images for: De novo MYC addiction as an adaptive response of cancer cells to CDK4/6 inhibition
Source: Mol Syst Biol. 2017 Oct 4;13(10):940. doi: 10.15252/msb.20167321 (PMC5658703; doi:10.15252/msb.20167321)

# Figure EV4.A

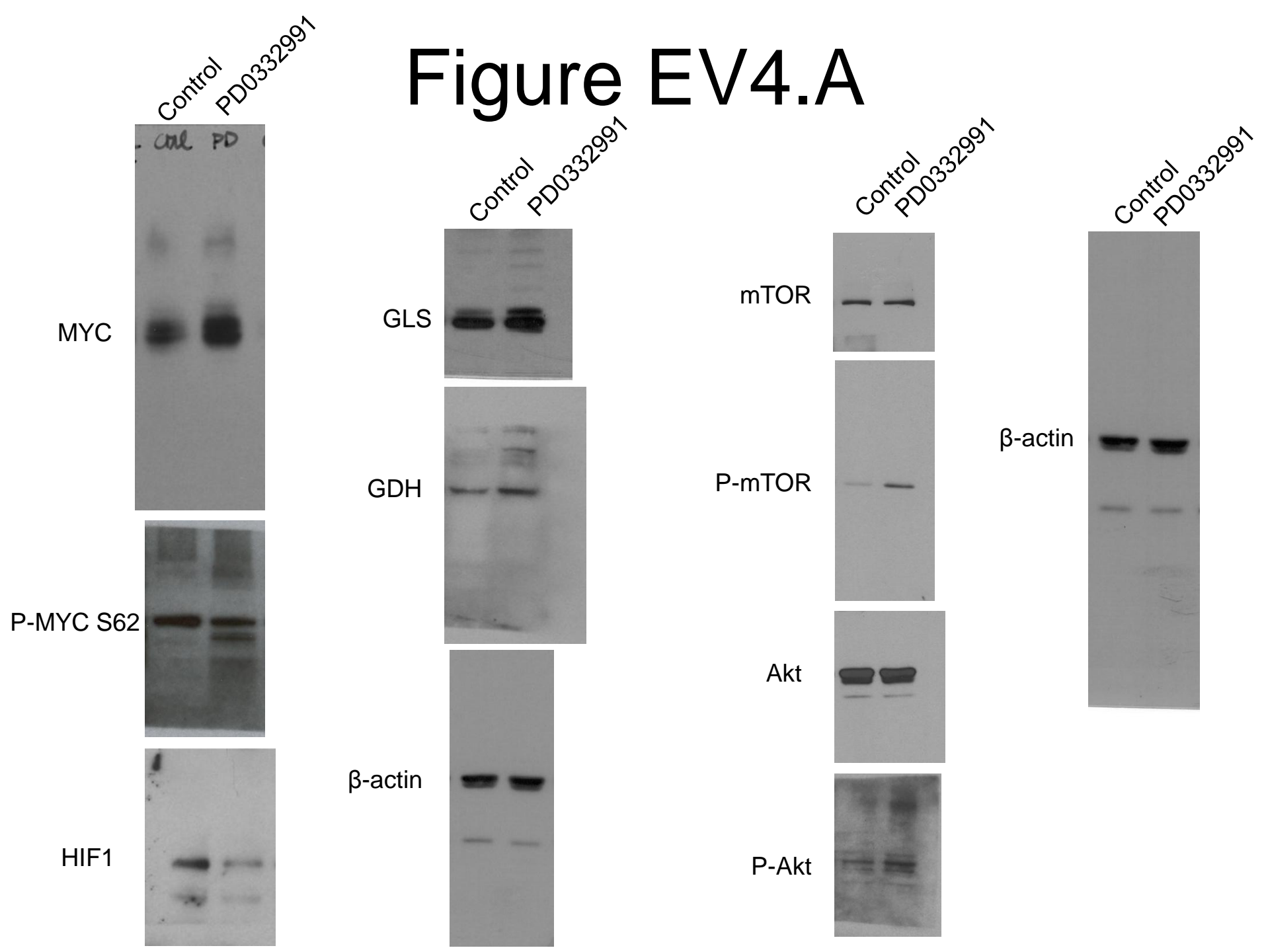

# Figure EV4.B

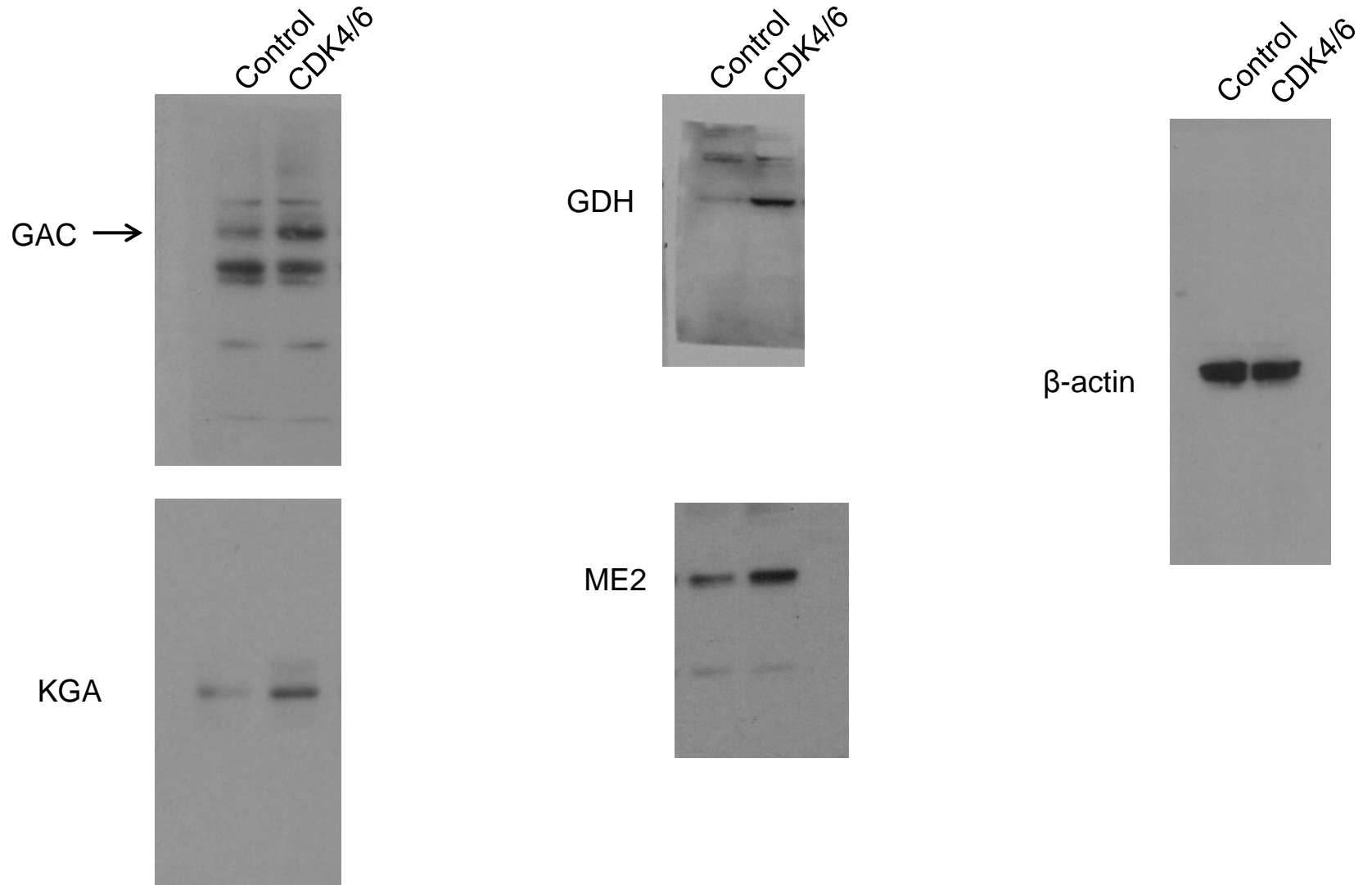

# Figure EV4.F

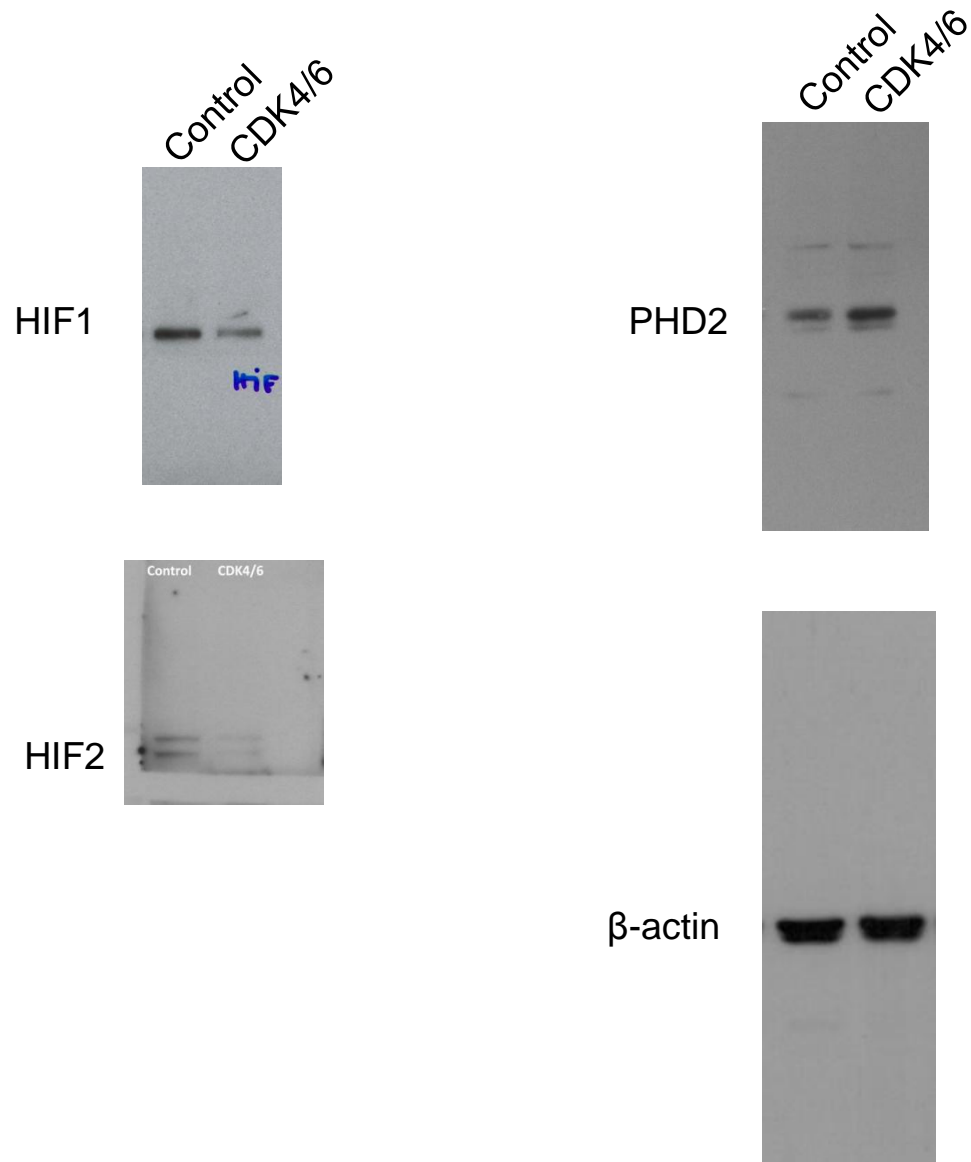

# Figure EV4.H

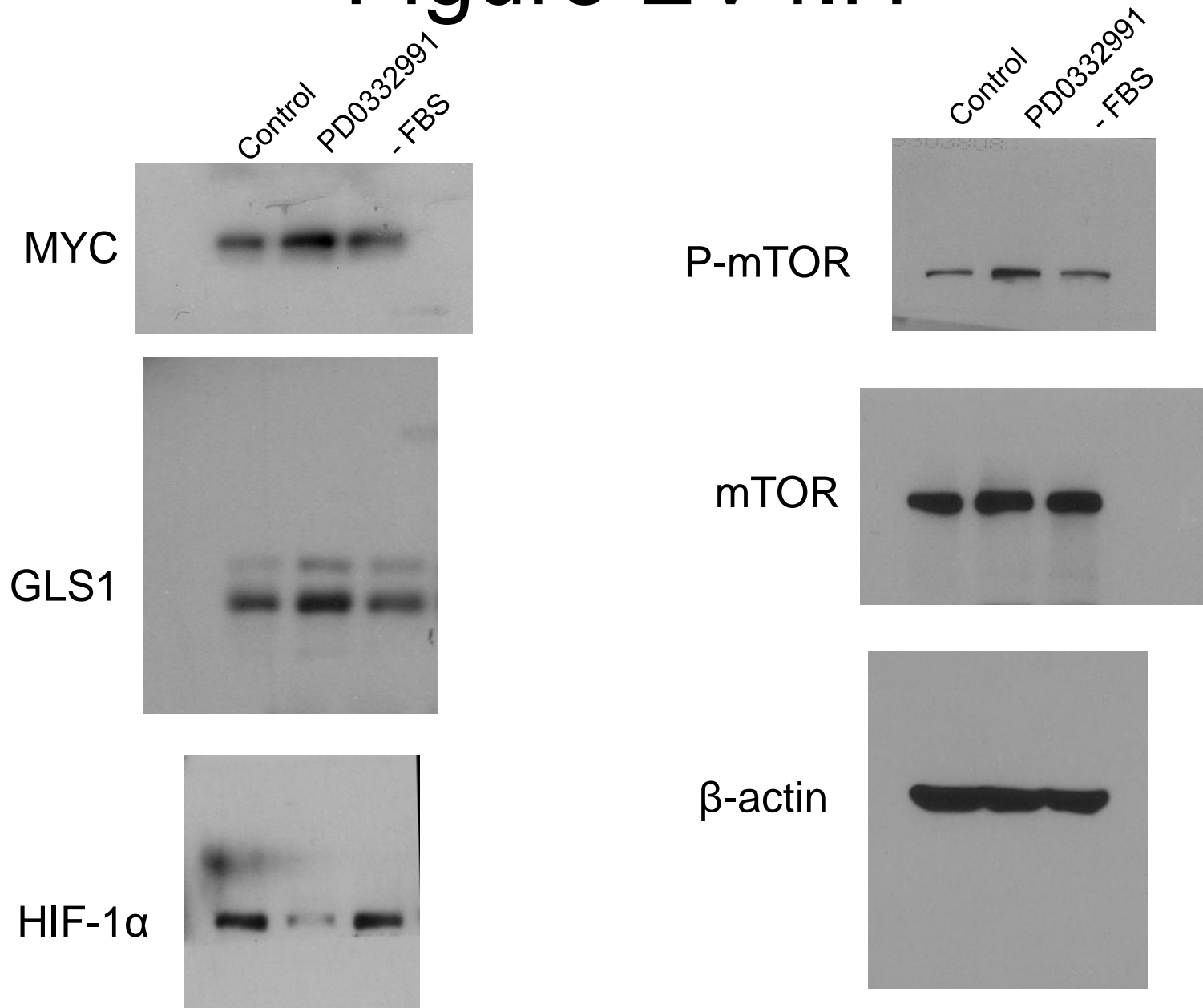

Supplement: Supplementary file 7 — Source Data for Expanded View [file MSB-13-940-s010.pdf]

# Figure 1.A

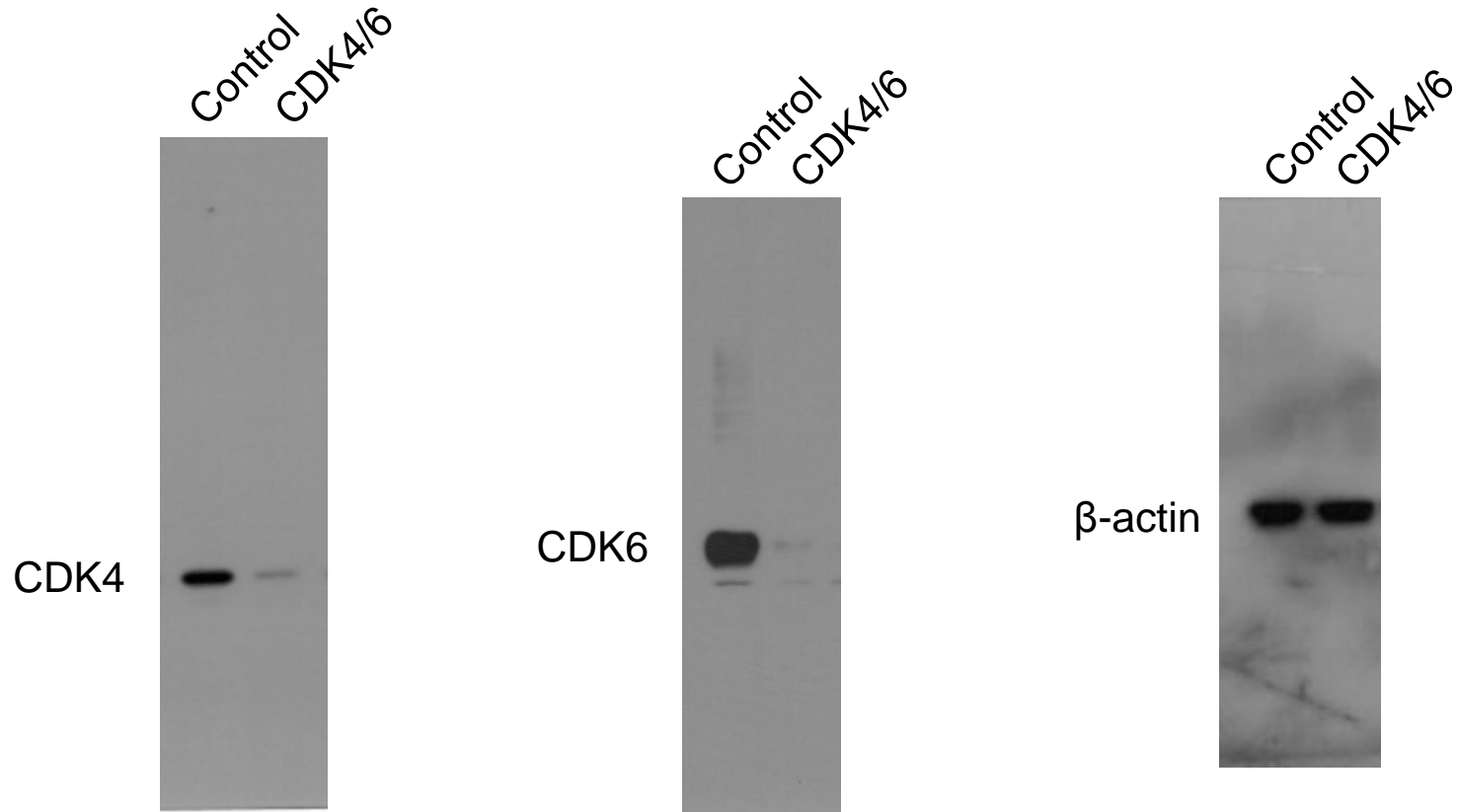

Supplement: Supplementary file 9 — Source Data for Figure 1 [file MSB-13-940-s007.pdf]

# Figure 5.A

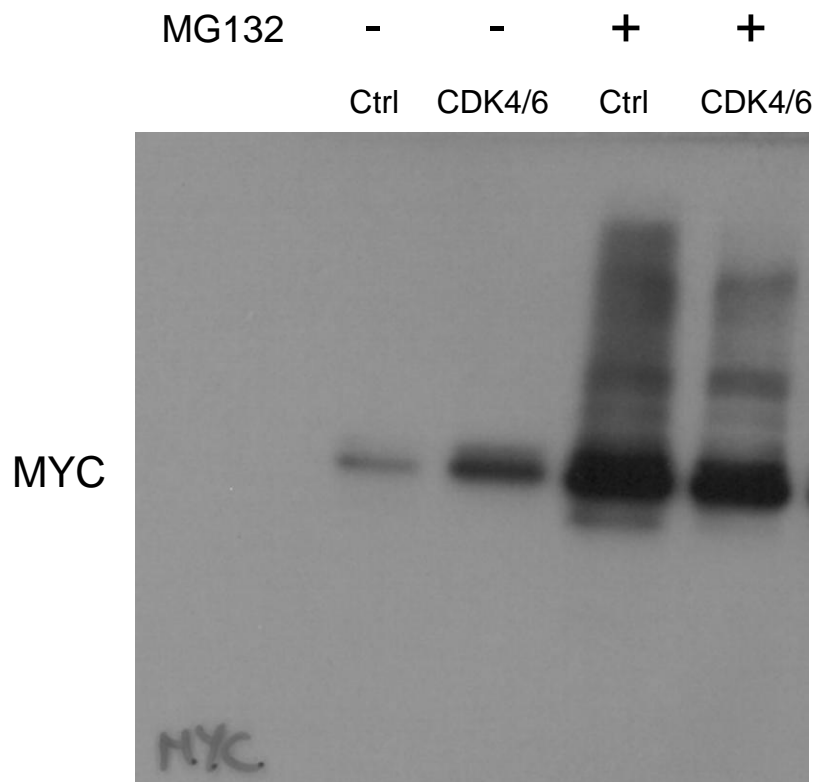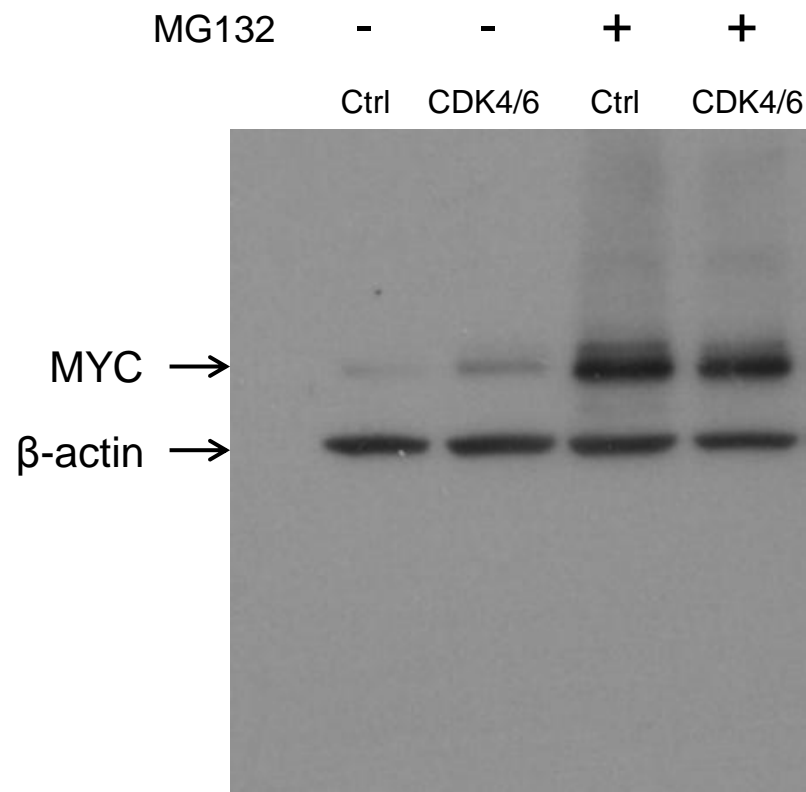

# Figure 5.C

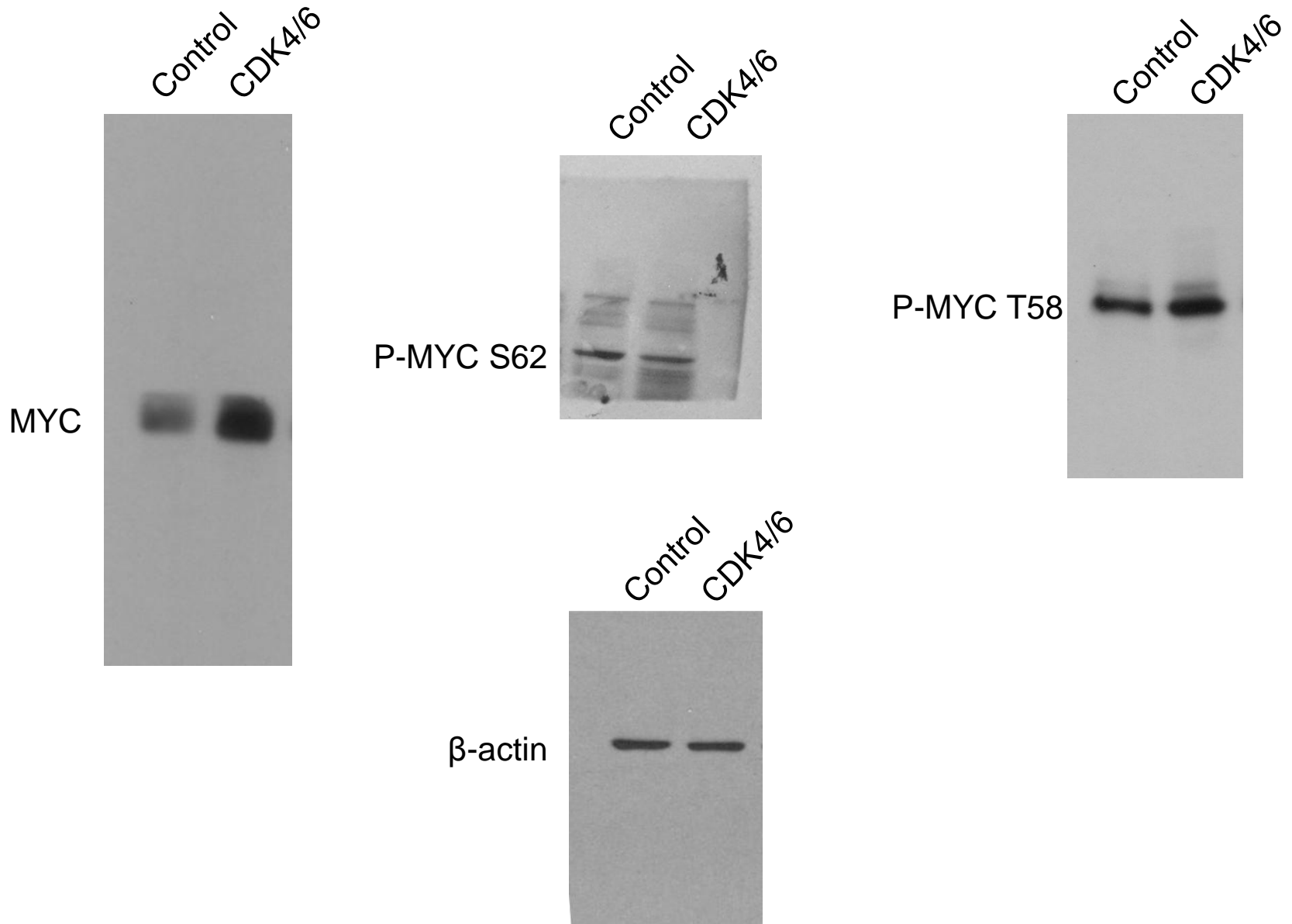

# Figure 5.E

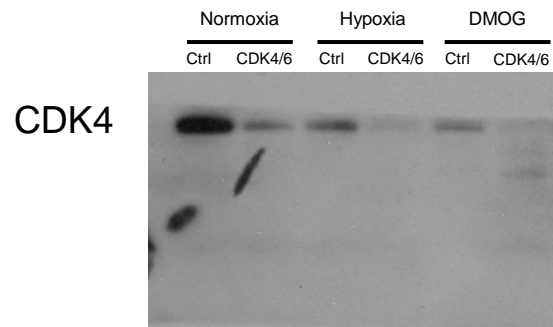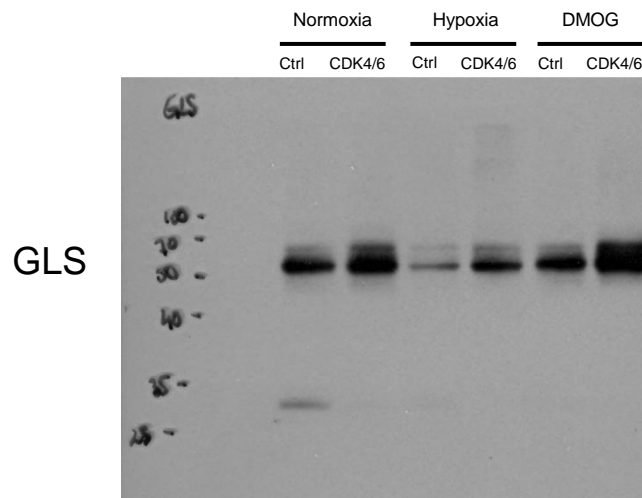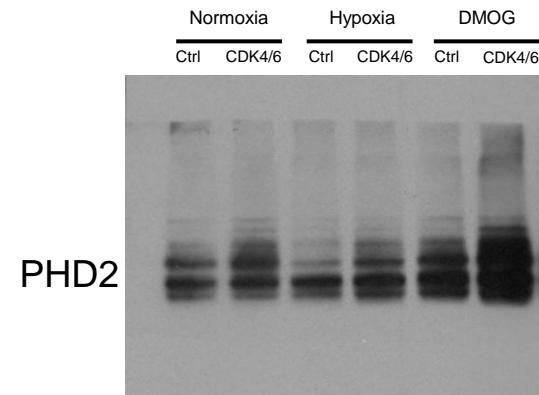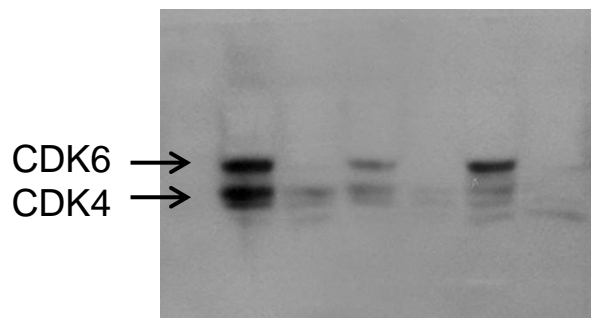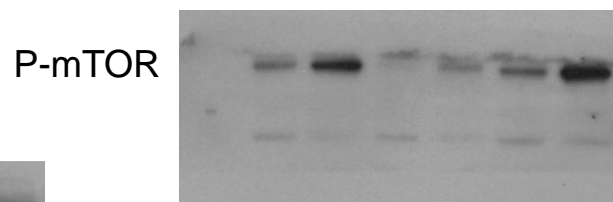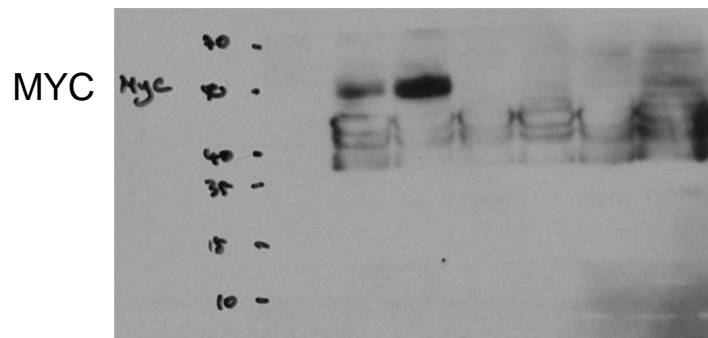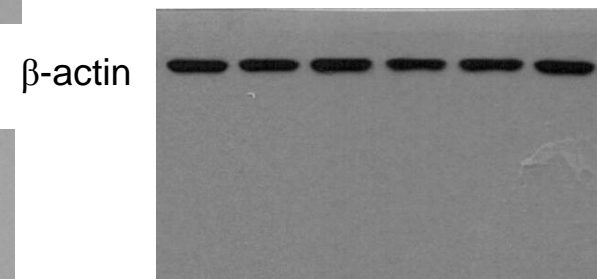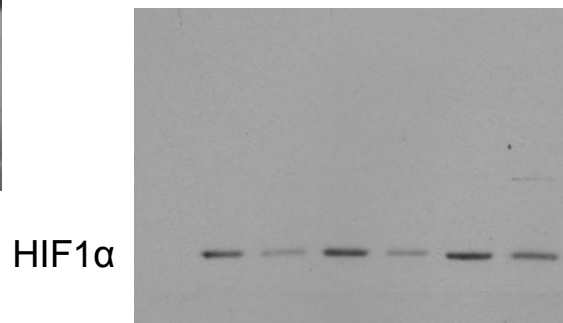

# Figure 5.G

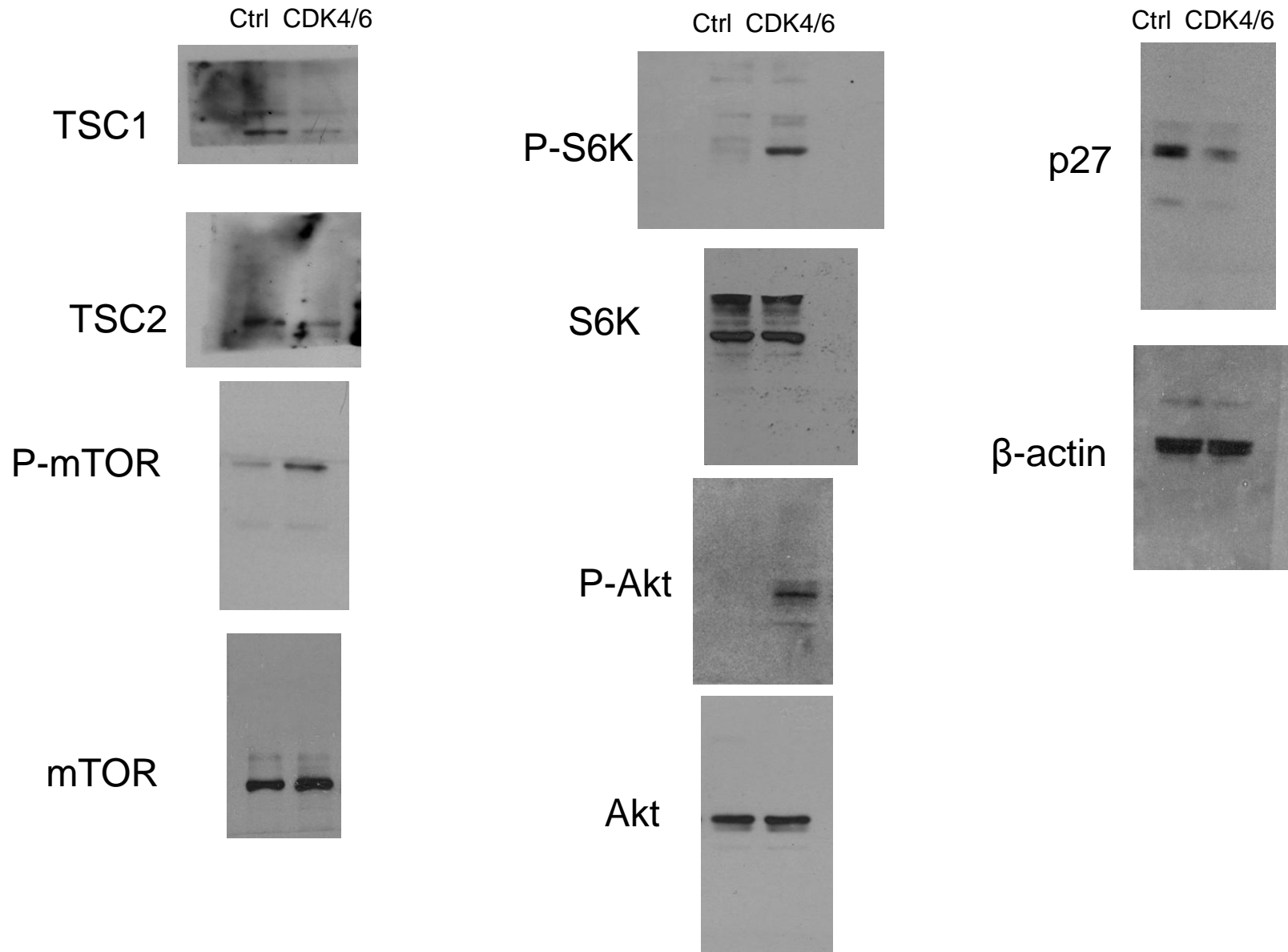

# Figure 5.H

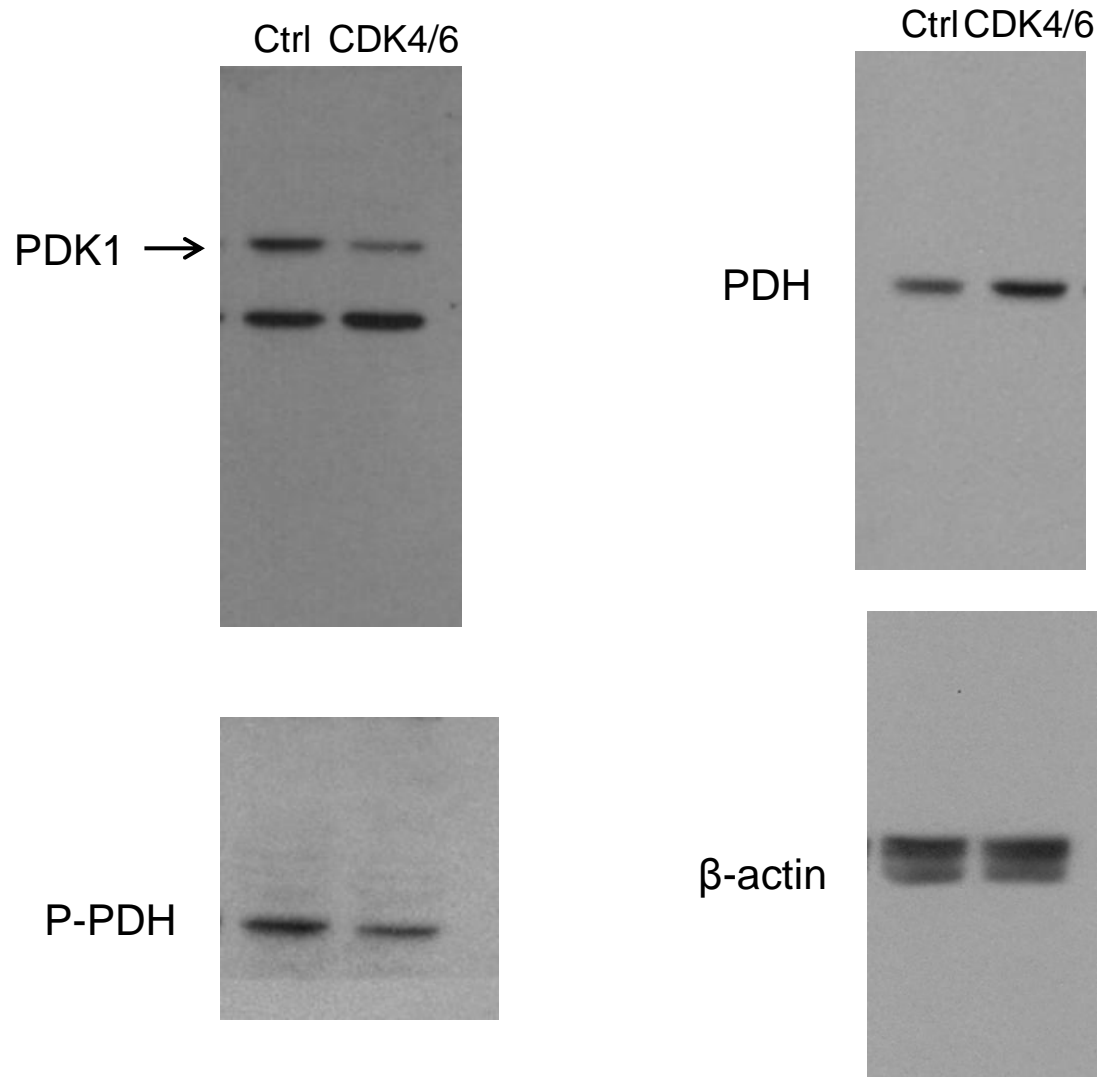

# Figure 5.J

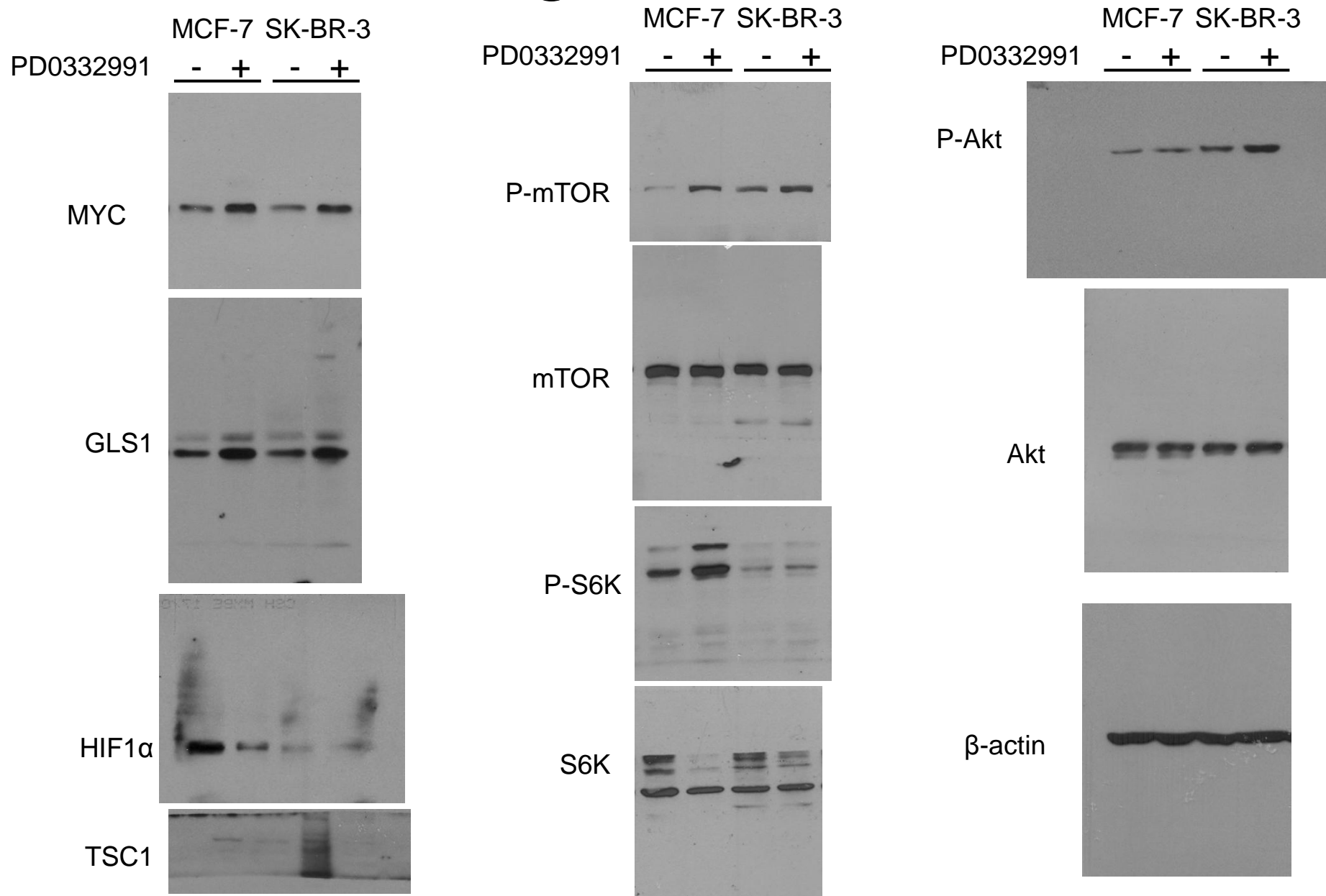

# Figure 5.L

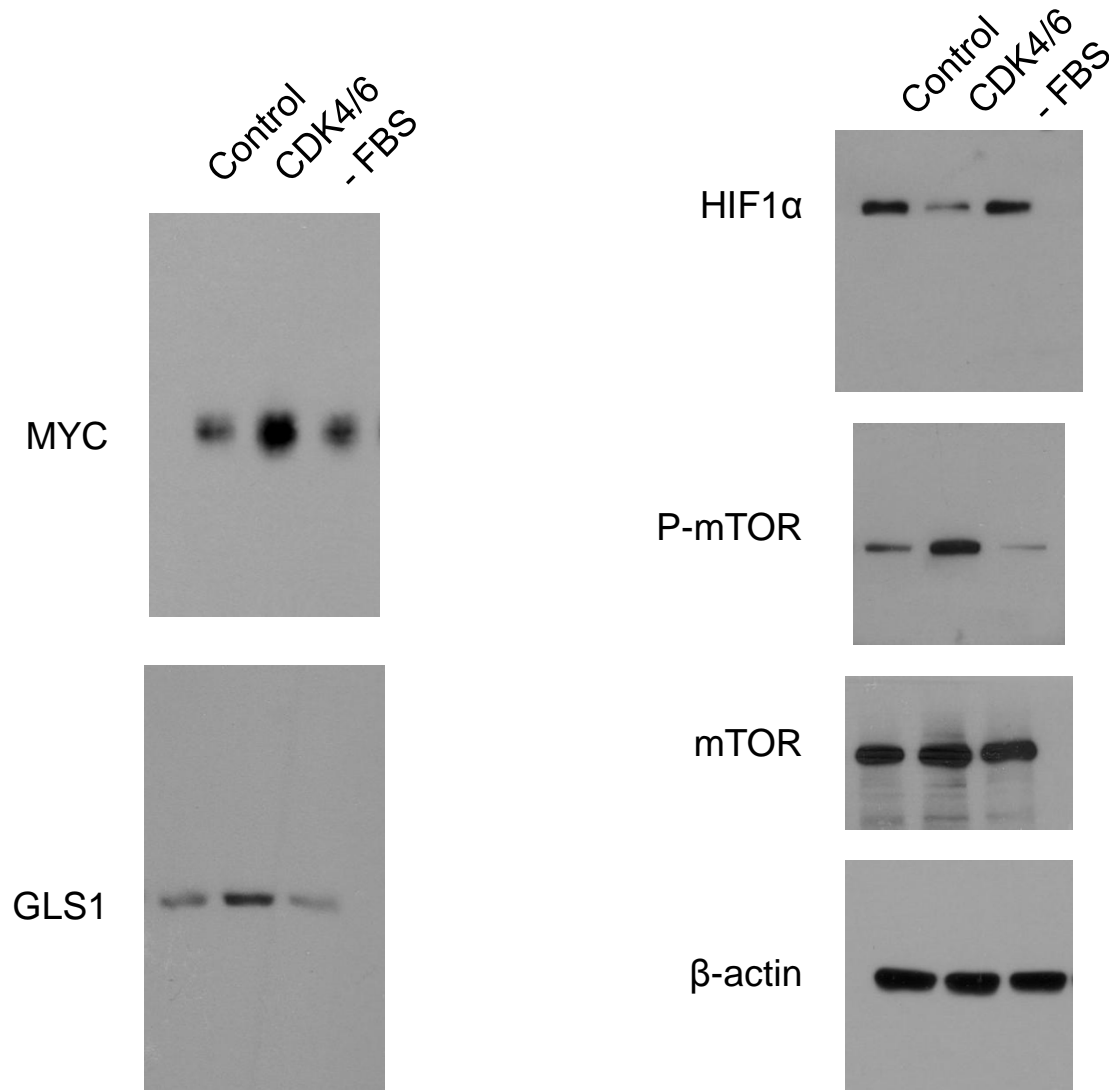

Supplement: Supplementary file 10 — Source Data for Figure 5 [file MSB-13-940-s008.pdf]

# Figure 6.A

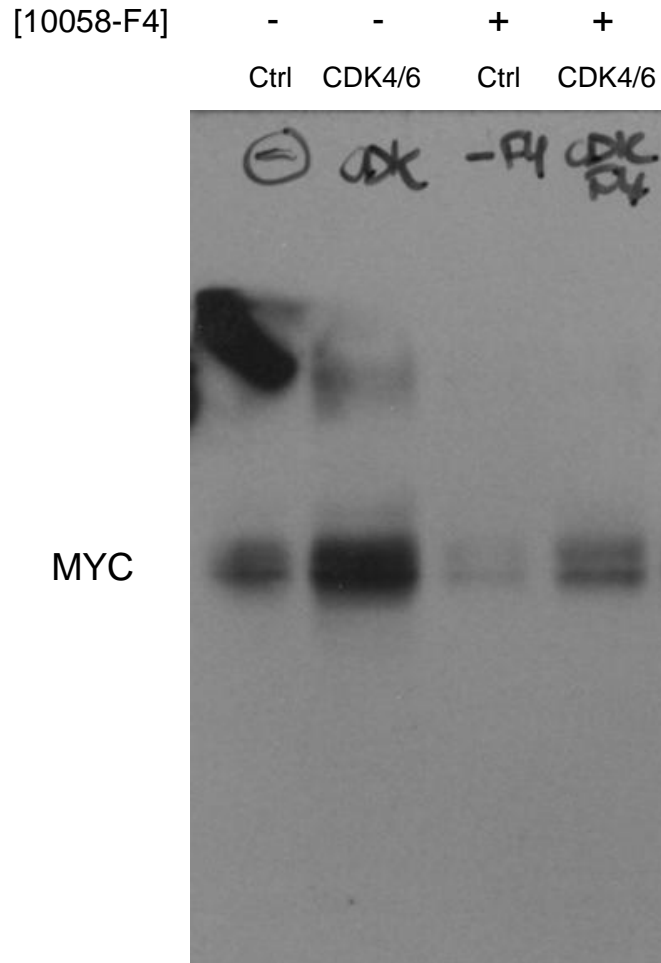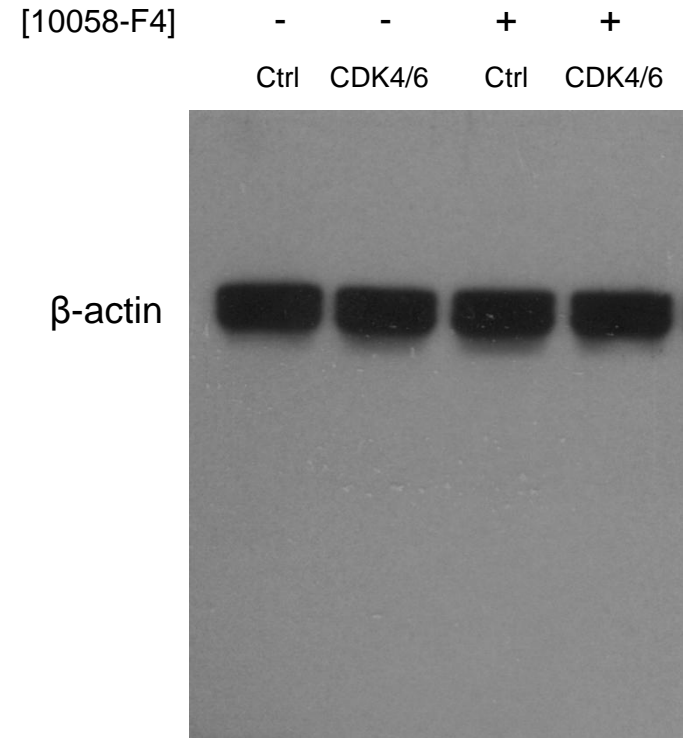

# Figure 6.B

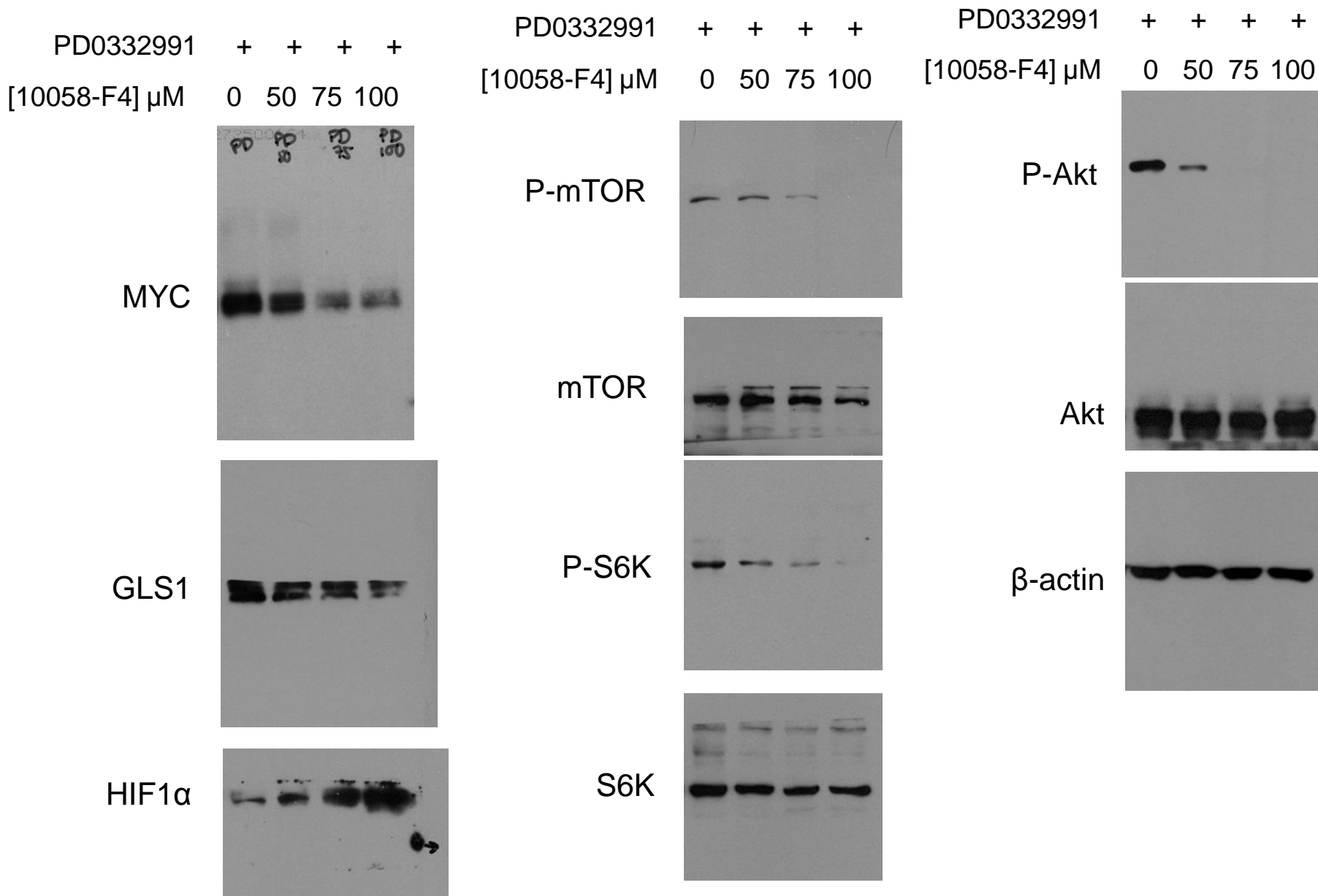

# Figure 6.E

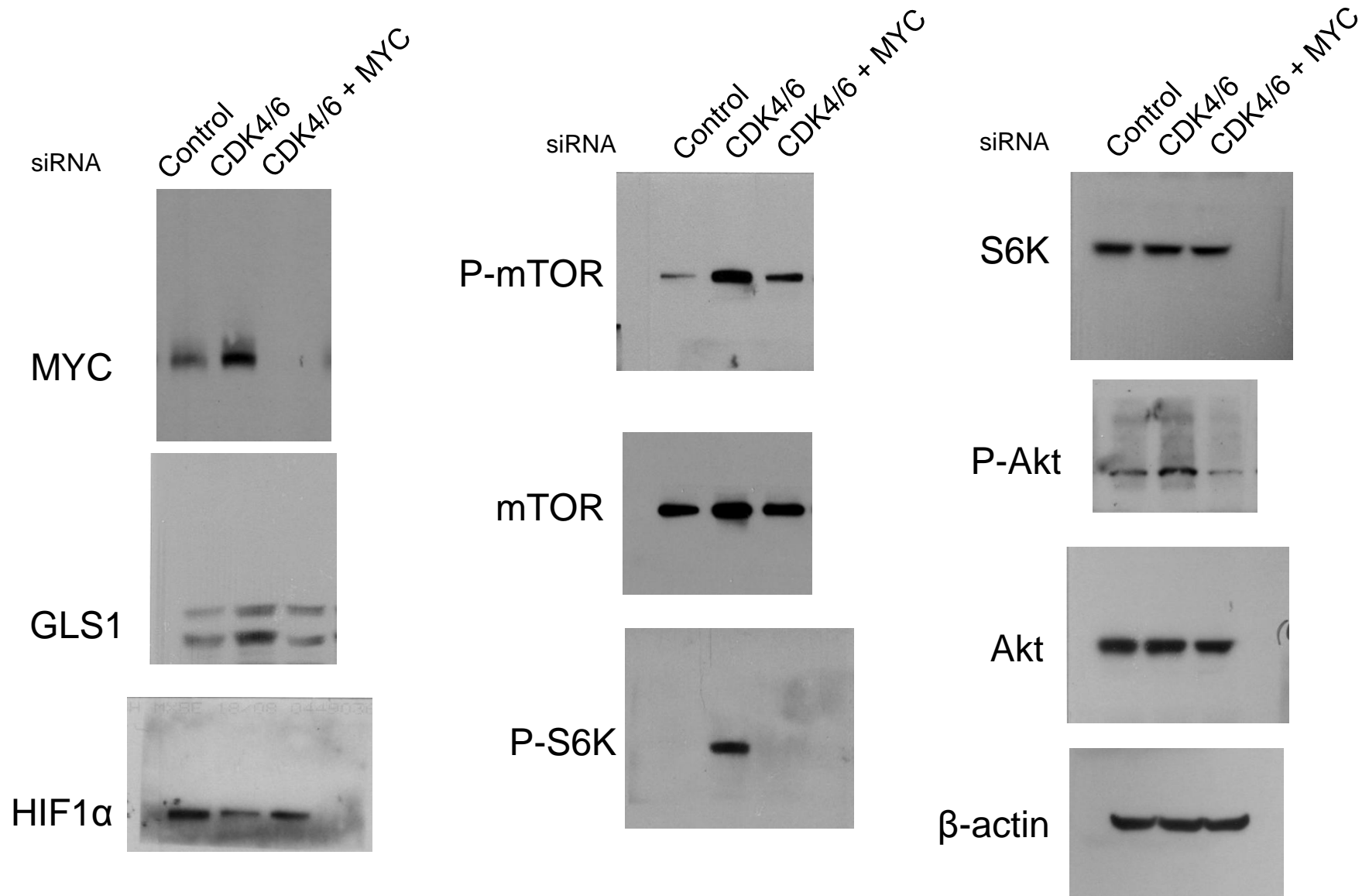

Supplement: Supplementary file 11 — Source Data for Figure 6 [file MSB-13-940-s009.pdf]
